# Cell membranes targeted unimolecular prodrug for programmatic photodynamic-chemo therapy

**DOI:** 10.7150/thno.55014

**Published:** 2021-01-19

**Authors:** Jie Yuan, Rong Peng, Dongdong Su, Xingxing Zhang, Hepeng Zhao, Xiujuan Zhuang, Mei Chen, Xiaobing Zhang, Lin Yuan

**Affiliations:** 1 State Key Laboratory of Chemo/Biosensing and Chemometrics, College of Chemistry and Chemical Engineering, Hunan University, Changsha 410082, P. R China.; 2 Department of Chemistry and Chemical Engineering, Beijing University of Technology, Beijing, 100124, P. R. China.; 3 College of Physics and Microelectronics Science, Hunan University, Changsha 410082, P. R China.; 4 College of Materials Science and Engineering, Hunan University, Changsha 410082, P. R China.

**Keywords:** cell membrane, singlet oxygen, glutathione, two-photon photodynamic therapy, combination therapy

## Abstract

Photodynamic therapy (PDT) has emerged as one of the most up-and-coming non-invasive therapeutic modalities for cancer therapy in rencent years. However, its therapeutic effect was still hampered by the short life span, limited diffusion distance and ineluctable depletion of singlet oxygen (^1^O_2_), as well as the hypoxic microenvironment in the tumor tissue. Such problems have limited the application of PDT and appropriate solutions are highly demand.

**Methods:** Herein, a programmatic treatment strategy is proposed for the development of a smart molecular prodrug (**D-bpy**), which comprise a two-photon photosensitizer and a hypoxia-activated chemotherapeutic prodrug. A rhodamine dye was designed to connect them and track the drug release by the fluorescent signal generated through azo bond cleavage.

**Results:** The prodrug (**D-bpy**) can stay on the cell membrane and enrich at the tumor site. Upon light irradiation, the therapeutic effect was enhanced by a stepwise treatment: (i) direct generation of ^1^O_2_ on the cell membrane induced membrane destruction and promoted the **D-bpy** uptake; (ii) deep tumor hypoxia caused by two-photon PDT process further triggered the activation of the chemotherapy prodrug. Both *in vitro* and *in vivo* experiments, **D-bpy** have exhabited excellent tumor treatment effect.

**Conclusion:** The innovative **programmatic** treatment strategy provides new strategy for the design of follow-up anticancer drugs.

## Introduction

Photodynamic therapy (PDT) has become one of the most promising methods for cancer treatment and attracted numerous attentons in recent years, due to its pentiful advantages, such as non-invasiveness, fewer side effects, good selectivity, and spatiotemporal responsiveness 1-3. However, great chanllenges still exsit in the cancer treatment with PDT. On one hand, singlet oxygen (^1^O_2_) as the main toxic substance of PDT, has extremely short lifetime (< 4 μs) and limited diffusion distance (< 20 nm), therefore, it can not be fully utilized unless it is generated nearby biological macromolecules 4, 5. On the other hand, ^1^O_2_ can be easily depleted by abundant glutathione (GSH, concentration range from 1 mM to 15 mM) exsisting in cancer cells 6, 7, which dramatically reduce the photodynamic effect, and ultimately, leading to a poor therapeutic effect 8-12. In this regard, many efforts have been made to solve this problem, and most of the studies were focused on reducing the content of GSH in cells to enhance the PDT effect 13-16. However, unfortunately, owing to the high GSH content in cancer cells, achieving satisfactory effects only by reducing the GSH content is extremely difficult. Therefore, in order to greatly improve the PDT efficiency, it is imperative to seek a strategy to simultaneously solve the two problems mentioned above.

Plasma membrane separates the interior of the cell from the outside environment and is enriched by many important proteins, its integrity determines the activity of the cells 17-19. Moreover, it was found that the concentration of GSH in cell membrane is negligible 7, 20. Such two characteristics of cell membrane inspired us that if we can design a photosensitizer that can remain on cell memebrane, it is possible to guarantee sufficient interaction of singlet oxygen with active proteins by the following two aspects. First, the distance between singlet oxygen and proteins can be tremendously shortened. Second, the consumption of singlet oxygen by GSH can be avoided due to its extreme low concentration. Thus, such photosensitizers are speculated to have great effect on destroying the cell membrane and affecting the cell activity.

Since the PDT process consumes oxygen in the tumor tissue, it will lead to a very severe degree of tumor hypoxia, and in turn inhibit the subsequent phototherapy process, ultimately, resulting in poor therapeutic effect 3, 21. Currently, many studies have been done to combine chemotherapy with photodynamic therapy to enhance the anti-cancer effect of PDT 22-29, and a certain extent of enhancement have indeed been achived. However, for some of these drug, the release process are based on the consumption of ^1^O_2_ generated during PDT, which still weaken the therapeutic effect 26, 30.

Combining the above points, it is greatly meaningful to create a multi-functional photosensitizer, which can not only stay on the cell membrane, but also release chemotherapeutic drugs controllably during the phototherapy without consuming ^1^O_2_, to realize the synergistic treatment of phototherapy and chemotherapy. In this work, we report the design and synthesis of a unimolecular prodrug (named as **D-bpy**), which can be enriched in the cell membrane and tumor tissues, and be cascade activated for programmatic cancer photodynamic-chemo therapy. The result showed that, comparing with the photosensitizer (**Bpy**) or chemotherapy prodrug (**R-drug**), **D-bpy** has exhabited a better therapeutic effect in living mice through the combination of two-photon PDT and chemotherapy.

## Methods

### Materials, cell culture, animals

All reagents used were purchased from commercial suppliers without further purification and solvents were purified by standard methods prior to use. For the synthesis experiment parts, thin layer chromatography (TLC) analysis was performed on silica gel plates and column chromatography was conducted over silica gel (mesh 200-300), both of which were obtained from the Qingdao Ocean Chemicals. Twice-distilled water was used throughout all experiments. HeLa and 4T1 cells were cultured in high glucose Dulbecco's Modified Eagle Medium (DMEM) supplemented with 10% fetal bovine serum (FBS), and 1% antibiotics (100 U/mL penicillin and 100 µg/mL streptomycin) at 37 °C and 5% CO_2_. Cells were carefully harvested and split when they reached 80% confluence to maintain exponential growth. Eight- to ten-week-old BALB/c female mice (body weight 16 ~ 18 g) were purchased from Hunan SLAC Laboratory Animal Co., Ltd. The mice were fed with distilled water and standard fodder at room temperature. The 4T1 murine breast tumor models were generated by subcutaneous injection of 50 µL phosphate buffer solution (PBS) containing 5 × 10^6^ cells onto the right rear flanks of each mouse. All animal experiments were performed in compliance with the relevant laws and approved via the institutional committee of Hunan University.

### Characterizations

Mass spectra were performed using Matrix Assisted Laser Desorption Ionization Time of Flight Mass Spectrometry (ultrafleXtreme). NMR spectra were recorded on a Bruker-400 spectrometer, using TMS as an internal standard. Absorption and fluorescence spectroscopic studies were performed on a UV 1800 ultraviolet and visible spectrophotometer and a HITACHI F4600 fluorescence spectrophotometer, respectively. The fluorescence images were acquired with a two photon confocal laser scanning microscope (Nikon, Japan) or an Olympus FV1000 equipped with a CCD camera; Inverted microscope images of calcein-AM and PI stained HeLa cells use AXIO system, ZEISS; The *in vivo* (living mice) imaging was carried out using an IVIS Lumina XR (IS1241N6071)* in vivo* imaging system. Apulsed laser at 800 nm were generated by a mode-locked Ti: sapphire laser (Tsunami) operating at 800 nm (pulse width 100 fs, repeated frequency 80 MHz) amplified by a regenerative amplifier laser (Spitfire Ace 100, 1 kHz).

### Synthesis of D-bpy

The smart molecular prodrug **D-bpy** was synthesized through four steps of reactions and the synthetic route is shown in **Scheme S1A**. As the control compound **R-drug** was obtained by a one-step acetylation of compound **3**, and **Bpy** was synthesized according to the previous literature (**Scheme S1B**) 31. All compounds were fully characterized by NMR and mass spectrum (see supporting information).

### Cell membrane colocalization assay

HeLa cells were plated onto confocal dishes for 24 h. Next, the cells were firstly incubated with **D-bpy** (5 μM) for 1 h at 37 °C under 5% CO_2_ consideration, and then stained by **Dio** (10 μM) for another 30 min. Cells were then washed with PBS and visualized with laser confocal microscopy. The excitation wavelength for **D-bpy** and **Dio** were 488 nm. The emission wavelength was collected from 620 to 680 nm for **D-bpy** and 500 to 530 nm for **Dio**.

### Cell membrane destruction experiment

HeLa cells were plated onto confocal dishes for 24 h. Next, the cells were divided into three groups, group one and group two were incubated with **D-bpy** (5 μM) for 1 h at 37 °C under 5% CO_2_ conditions, and then stained by **Dio** (10 μM) for another 40 min. Group three was incubated with **Dio** (10 μM) for 40 min only. During the visualization with laser confocal microscopy, group one and group three were continuously illuminated with low-energy LED light (450-470 nm) while group two without illumination. The excitation wavelength for **D-bpy** and **Dio** were 488 nm. The emission wavelength was collected from 620 to 680 nm for **D-bpy** and 500 to 530 nm for **Dio**.

### Fluorescence imaging of rhodamine green in tumor tissues after PDT

The 4T1 tumor-bearing mice were injected intratumorally with **D-bpy** firstly, and then randomly divided into two groups (three mice in each group). After 2 h, the mice of group one were treated with 800 nm TP laser (0.8 W/cm^2^, 15 min) for photodynamic therapy and the other group was not. After 16 h of treatment, the mice were sacrificed and the tumor tissue were then excised for imaging, the excitation wavelength was 488 nm, and emission wavelength was collected from 500 to 550 nm.

### *In vivo* tumor accumulation

**D-bpy** (100 μL, 500 μM) and **Bpy** (100 μL, 500 μM) was injected intravenously into tumor bearing mice, respectively, and the mice were randomly divided into ten groups, each group contained three mice. Fluorescence imaging was observed at different post-injection time. After 0 h, 3 h, 6 h, 10 h and 32 h post-injection, respectively, and the mice were sacrificed. Then, the main tissues and tumor tissues were taken for *ex vivo* imaging. The excitation wavelength was 465 nm, and the collected emission wavelength was 575-650 nm.

### *In vivo* PDT efficacy of D-bpy

In order to compare the *in vivo* PDT efficacy of **D-bpy**, **Bpy** and **R-drug**, the mice bearing 4T1 tumor were divided into eight groups randomly, each group contained four mice and performed with the following different treatments: group 1, PBS injection; group 2, PBS injection and irradiation; group 3, **D-bpy** injection; group 4, **D-bpy** injection and irradiation; group 5, **Bpy** injection; group 6, **Bpy** injection and irradiation; group 7, **R-drug** injection; group 8, **R-drug** injection and irradiation. **D-bpy** (20 μL, 500 μM), **Bpy** (20 μL, 500 μM) and **R-drug** (20 μL, 500 μM) were injected through intratumor injection subcutaneously. After 2 h post-injection, tumor region was irradiated with 800 nm TP laser at a power density of 0.8 W/cm^2^ for 15 min. After 15 days post-treatment, the mice were euthanized, and tumor tissues of mice were harvested for weighing and tumors of each group were taken out at 16 h post treatment for H&E staining.

The *in vivo* PDT efficacy of **D-bpy** was also explored by intravenous injection (100 μL, 500 μM) using the same experimental procedure as intratumor injection. In addition, main organs of each group were collected at 15 days post treatment for hematoxylin and eosin (H&E) staining.

## Results and Discussion

### Design and synthesis of D-bpy

Generally, substances that contain both hydrophilic and hydrophobic parts may increase the chance of accumulating on cell membranes 17, 32-34. Except for hydrophobic interaction, electrostatic interaction can also act as the driving force for targeting cell membranes 35. In addition, comparing to one-photon (OP) excitation, two-photon (TP) excitation use a low-energy NIR laser as the light source, which has less tissue damage and deeper tissue penetration, and can be used with greater spatial precision which is beneficial for the treatment of deep tumors 28, 36-42. Thus, in this study, a two-photon photosensitizer ruthenium(II) polypyridyl complex with two positive charges was chosen as the hydrophilic moiety due to its good photochemical stability, good two-photon properties, high singlet oxygen yield, low dark toxicity, and good hydrophilicity 36-38, 43-46. For the hydrophobic moiety, a fat-soluble prodrug was designed by attaching a widely used alkylating agent that can induce cell death by disrupting nuclear DNA to the rhodamine dye using for tracking the release of chemotherapy drug. Herein, an azo bond was used to connect the alkylating agent and the dye, since azobenzene chromophore can be reduced efficiently by azoreductase in anoxic microenvironment and has been widely used in hypoxia detection and drug release 47-53. In this case, it can make full use of the deep hypoxia caused by PDT to promote azo bond breakage, and then release chemotherapeutic drug to a greater extent, so as to achieve the purpose of synergistic treatment of phototherapy and chemotherapy. Furthermore, because the surface of cancer cells has more negative charges than normal cells, positive-charged molecules are prone to be enriched in tumor cells 17, 54, 55. Therefore, **D-bpy** is expected to have the structure-inherent tumor-accumulating ability. We expect that **D-bpy** can be first enriched in the tumor tissue and anchor on the cellular membrane, then the programmatic treatment is triggered by photodynamic therapy, which further drives the activation of chemotherapy, resulting in the chem/photo-dynamic combined therapy. And in the dark condition, **D-bpy** could stay on the cell membrane with limited cellular internalization, which greatly reduces the side effects of the chemotherapeutic prodrug.

**Figure 1:**
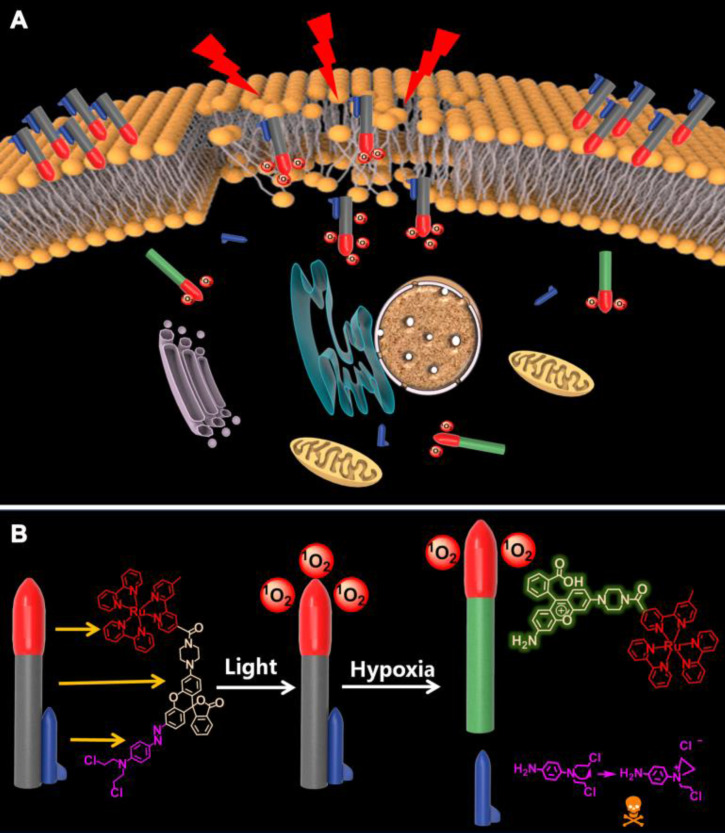
** Schematic illustration of photoactivatable cancer treatment of D-bpy.** (A) Illustration of the combined treatment of **D-bpy** upon light irradiation. (B) Structure and mechanism of action of **D-bpy**.

The “trilogy” mechanism of **D-bpy** during treatment process can be described as follows: i) **D-bpy** accumulates on the cancer cell membrane first, and subsequently, the membrane lipid will be peroxidized by ^1^O_2_ generated from **D-bpy** under light irradiation, resulting in extreme tumor hypoxia and cell membrane damage. ii) **D-bpy** will enter the cells freely through the plasma membrane destruction. iii) The alkylating agent prodrug will be liberated into cells by breaking the azo bond under anoxic conditions to further promote the cell death. The specific mechanism is shown in **Figure 1**.

### ^1^O_2_ generation and drug release *in vitro*

**Figure 2:**
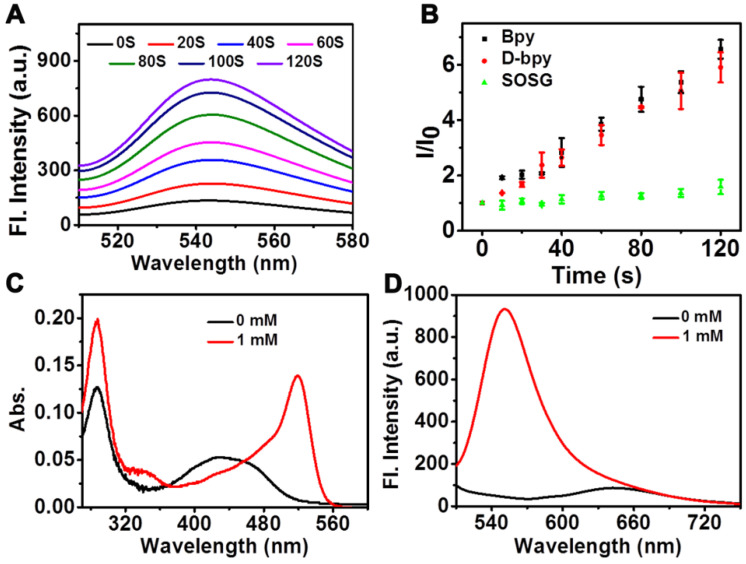
**^1^O_2_ production and drug release test *in vitro*.** (A) Fluorescent spectra of the mixture of **D-bpy** and SOSG upon irradiation. (B) Measurement of ^1^O_2_ production efficiency via changes in the fluorescence by SOSG at 540 nm versus irradiation time (irr = 450-470 nm) in the presence of adjusted concentrations of **D-bpy** and Bpy in MeOH. The absorption (C) and fluorescence intensity (D) of **D-bpy** (5 µM) reacted with sodium dithionite in PBS (1% DMSO, pH = 7.4) for 20 min at 37 °C. λ_ex_ = 488 nm.

To verify the function of **D-bpy**, the ability of **D-bpy** to generate ^1^O_2_ was first explored. We chose the singlet oxygen sensor green (SOSG) as the ^1^O_2_ indicator to test ^1^O_2_ generation ability of **D-bpy**. As shown in **Figure 2A** and **Figure S14**, under 450-470 nm LED light irradiation, a significant increase in fluorescence at 540 nm was observed in the mixture of the SOSG and **D-bpy** solution, indicating the generation of ^1^O_2_. The ability of ^1^O_2_ generation of **D-bpy** and [Ru(bpy)_3_]^2+^(**Bpy**) was also compared (**Figure 2B**). The results showed that the modified prodrug **D-bpy**, maintaned the good photodynamic effect from the maternal photosensitizer. The singlet oxygen yield (Φ_△_) of **D-bpy** was also determined by using 1,3-diphenyl-isobenzofuran (DPBF) as the ^1^O_2_ detection reagent in methanol (**Figure S15**), and the value was calculated to be 77%, using **Bpy** (Φ_△_ = 81%) as a reference 38. Next, the reduction and cleavage behaviors of the azo double bond were tested by reacting with sodium dithionite and monitoring the changes in the ultraviolet-visible (UV/vis) absorption and fluorescence spectra (**Figure 2C-2D**). The conversion from aniline of rhodamine dye into azo derivative will lead to the closed lactone form, inducing the rhodamine dye had almost no signals at its maximum excitation and maximum emission peaks. What's more, a rotation mechanism around the -N=N- double bond or an inversion mechanism can further promote the quenching effect 56. When sodium dithionite was added, the azo bond broke and induced strong signals appeared at the characteristic peaks of UV/vis absorption and fluorescence of rhodamine dye. The reduction of azo bond was also confirmed by matrix-assisted layer desorption/ionization-time-of-flight mass spectrometry (MALDI-TOF/MS) (**Figure S12**) analysis. And the HPLC chromatogram also clearly verified the release of aniline nitrogen mustard (**Figure S13**). We also irradiated **D-bpy** for 1 h and no change in the UV absorption spectrum was observed (**Figure S16**), the result confirmed that the azo bond of **D-bpy** is stable and would not break under light conditions in normoxia. Subsequently, a simulation of the azo bond release under anoxic conditions was performed (**Figure S17**). It can be clearly seen that the fluorescence signal of the rhodamine derivative increased significantly under the hypoxic conditions, indicating the breakage of the azo bond of **D-bpy** and the release of the alkylating agent. In addition, the drug release behaviors under different O_2_ concentrations was also studied (**Figure S18**), and the result showed that increased cell hypoxia can promote drug release.

**Figure 3:**
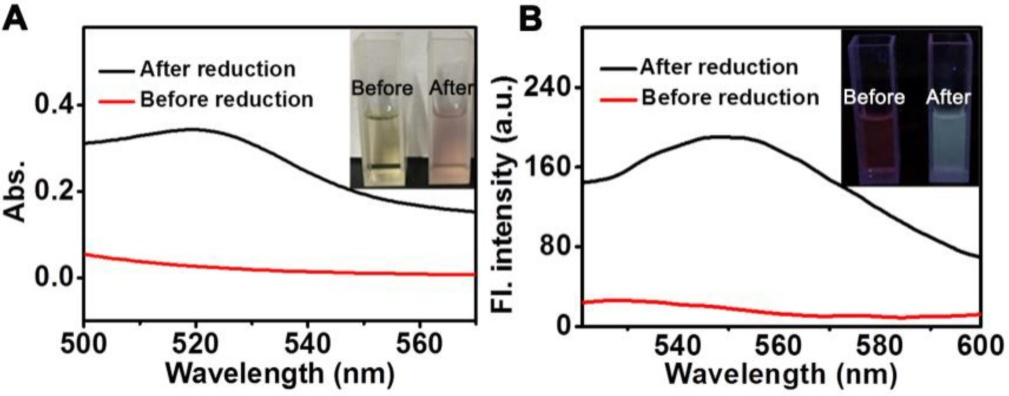
**Drug release under anoxic condition.** (A) UV-vis and (B) fluorescent spectra of **D-bpy** before and after reduction by rat liver microsomes and NADPH.

To evaluate the response of **D-bpy** in real biological systems, azoreductase-containing rat liver microsomes were used to cleave the azobenzene chromophores under hypoxic condition 53, and then the changes in the ultraviolet-visible absorption and fluorescence spectra were monitored (**Figure 3A, 3B**). We can see that an increase in fluorescence intensity at 550 nm was observed after incubated with rat liver microsomes and NADPH. This may be attributed to the breaking of azo bond caused by azoreductases in liver microsome, resulting in the ring opening and fluorescence recovery of rhodamine derivative.

**Figure 4:**
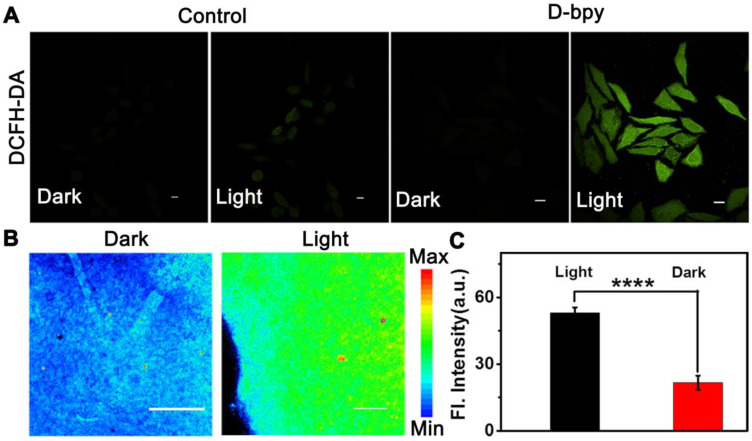
** Intracellular ^1^O_2_ production and drug release in living tissues.** (A) Confocal fluorescent images of HeLa cells incubated with **DCFH-DA** and **D-bpy** before and after TP irradiation. Scale bars = 10 µm. (B) Fluorescence images and (C) Mean fluorescence intensity of tumor tissues from BALB/c mice with 4T1 tumor after intratumoral injection of **D-bpy** with/without light irradiation. Scale bars = 25 μm. ****p < 0.0001.

Functional validation assays of **D-bpy** had also been studied in cells and tissues. Firstly, the two-photon absorption (TPA) and two-photon luminescence images in cells of** D-bpy** were conducted to choose the appropriate two-photon wavelength (**Figure S19-20**). Thereafter, 2,7-Dichlorofluoresceindiacetate (**DCFH-DA**), which transforms into strong green fluorescence by endogenous reactive oxygen species (ROS), was used as an indicator of ^1^O_2_ generation. As shown in **Figure 4A** and **Figure S21**, **D-bpy**, fortunately, exhibited improved ability to generate ^1^O_2_ to illumine **DCFH-DA** under OP or TP illumination. Similarly, we also used the singlet oxygen indicator SOSG to test the ^1^O_2_ generation ability of **D-bpy**, as shown in **Figure S22**, the cells incubated with the **D-bpy** and SOSG showed obvious green fluorescence after light irradiation, indicating that **D-bpy** had a good ability to produce ^1^O_2._ For tissue imaging experiment, BALB/c mice with 4T1 tumors were used and randomly divided into two groups, which were intratumorally injected with same dose of **D-bpy** (20 μL, 500 μM) for 2 h. An 800 nm two-photon laser was used for the deep tissue penetration to irradiate the tumor site for PDT. One group was illuminated for 15 min while the other group was not. After 16 h of treatment, the mice were sacrificed and tumors were excised for tissue imaging. As shown in **Figure 4B-4C**, the green fluorescence of rhodamine in the tumor tissue increased significantly after exposure to light, which perfectly demonstrated the rupture of the azo bond and the release of the alkylating agent drug.

### Co-localization and real-time imaging in cells with/without light irradiation

**Figure 5:**
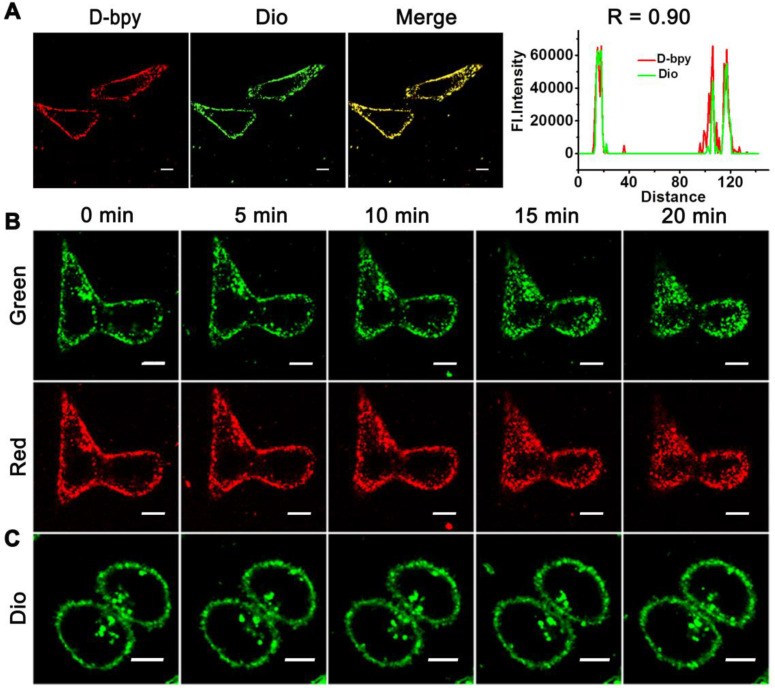
** Cell localization and real-time imaging of D-bpy and Dio in cells under different conditions.** (A) Colocalization images of **D-bpy** in HeLa cells. Cells were incubated with **D-bpy** (5 µM) for 1 h and then **Dio** (10 µM) was added and incubated for another 40 min. (Pearson's coefficient: R = 0.90) (B) Confocal images of living HeLa cells firstly incubated with **D-bpy** (5 µM) for 1 h and then **Dio** (10 µM) was added and co-incubated for another 40 min or (C) only incubated with **Dio** and then through continuous irradiation or without irradiation. Scale bars = 10 µm.

The lipophilicity/hydrophilicity of **D-bpy** and **Bpy** were conducted before performing the co-localization imaging 38. The octa-nol/water partition coefficient (log *P_o/w_*) of **D-bpy** was determined to be 3.57, which was more lipophilic than **Bpy** (log *P_o/w_* = -1.28) (**Figure S23**) due to the introduction of the fat-soluble prodrug. Therefore, with two positive charges and strong fat solubility, **D-bpy** has potential for cell membrane targeting because of the electrostatic interactions and hydrophobic interactions 35. Then the co-localization experiments were performed to verify the membrane targeting of **D-bpy** by using a commercial membrane localization reagent (**Dio**) as a control. As shown in **Figure 5A**, the channel signal of **D-bpy** (red channel) is highly coincident with that of **Dio** (green channel) and the co-localization coefficient reaches 0.90, indicating that **D-bpy** had a good membrane-targeting performance. In addition, we had verified this special property of the **D-bpy** with a variety of cells (**Figure S24**), and all results showed that the **D-bpy** had good membrane targeting ability. To this end, a continuous lighting experiment was performed to verify the rationality of our design, and to prove the initial ^1^O_2_ generated by **D-bpy** could subsequently lead to the destruction of the cell membrane structure and further promotes the entry of **D-bpy** into cells. The experiment included three groups (**Figure 5B-5C** and **Figure S25**): the first and second groups were incubated with **D-bpy** and **Dio**, while the third group was incubated with **Dio** only. After a period of time, the position changes of **D-bpy** and **Dio** in cells were obseved. The first and third groups were illuminated continuously, while the second group had no illumination. The results showed that **D-bpy** in the first group was transferred from the cell membrane to the inside of the cells (**Figure 5B**), while the position of **D-bpy** in the second (**Figure S25**) and third group (**Figure 5C**) did not change. These results indicated that the cell membrane only rupture when **D-bpy** and light coexist and further lead to the free entry of extracellular substances into the cell, suggesting the ^1^O_2_ generation by **D-bpy** and cause the cell membrane destruction, which is consistent with our expectations.

### *In vitro*/*vivo* treatment effect

**Figure 6:**
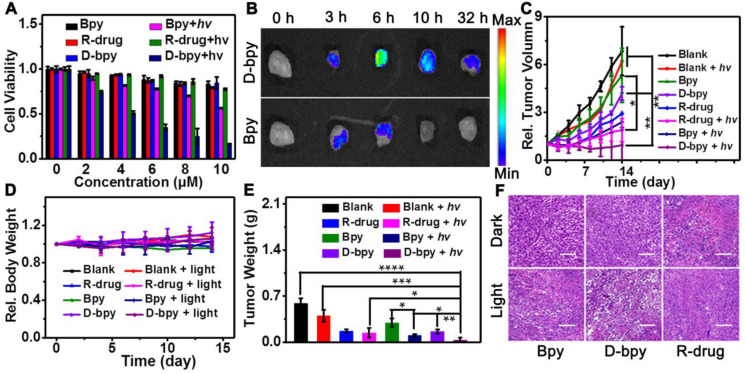
***In vitro* and *in vivo* therapeutic effect evaluation.** (A) Cell viability of **D-bpy**, **Bpy**, **R-drug** with/without light irradiation in HeLa cells. (B) Fluorescence imaging of tumor tissue from 4T1 tumor-bearing BALB/c mice after intravenous injection of **D-bpy** and **Bpy** respectively. (C) Relative tumor volume changes of mice with different treatments (*hv* = 800 nm). (D) Relative body weight of different treatments. (E) Tumor weight of the mice with different treatment. (F) H&E staining of tumors with different treatments after PDT. Scale bars = 100 μm. *p < 0.05, **p < 0.01, ***p < 0.001, ****p < 0.0001.

MTT colorimetric assay was performed using HeLa cells and 4T1 cells respectively to compare the inhibitory effects of **D-bpy**, **Bpy** and **R-drug** to inhibit cell growth simultaneously (**Figure 6A**,**Figure S26**). The experiments indicated that **D-bpy** had better cytotoxicity than **Bpy** and **R-drug** under light irradiation. The results are in accordance with our expectations and can be explained by the “programmatic mechanism” of **D-bpy**. Since **D-bpy** has a cell membrane-anchoring function, it can stay on the cell membrane without entering the cell in the absence of light, which greatly reduces the side effects of the prodrug. Under light irridiation, for **D-bpy**, the membrane lipids are first peroxidized by ^1^O_2_ generated on the cell membrane, resulting in very severe tumor hypoxia and cell membrane destruction. Thereafter, **D-bpy** freely passes through the cell membrane into the cell to initiate chemotherapy under anoxic conditions and thoroughly kills cancer cells through a combination of PDT and chemotherapy. Live cell/dead cell staining experiments further confirmed the enhanced intuitive nature of **D-bpy** as an ideal photosensitizer, that is, **D-bpy** was highly toxic when exposed to light but not toxic in the dark (**Figure S27**). In addition, we evaluated the therapeutic effects of the different treatments. The results showed that the **D-bpy** resulted in more cell deaths under light irradiation (**Figure S28**), indicating that the combined treatment had a better therapeutic effect than chemotherapy and photodynamic therapy alone.

The enrichment behavior of **D-bpy** and **Bpy** on tumors was investigated to verify the accumulation ability of **D-bpy** in tumors. **D-bpy** and **Bpy** were intravenously injected into BALB/c mice with 4T1 tumors, separately, to observe changes in the fluorescent signal of the main tissues and the tumor site over time (**Figure 6B**,**Figure S29**). As shown in **Figure 6B**, the fluorescence intensity of **D-bpy** reached its maximum at 6 h and did not disappear until 32 h. However, compared with **D-bpy**, the fluorescence signal of **Bpy** was much weaker at 6 h and completely disappeared at 10 h. Clearly, **D-bpy** has better tumor-targeting ability than **Bpy**. This may be due to the higher lipophilicity of **D-bpy**. Although both **Bpy** and **D-bpy** contain two positive charges, **D-bpy** becomes more lipophilic after modification, and lipophilic positive-charged molecules tend to be retained in the tumor site 17, 55, 57. To further prove that the change of fluorescence intensity at the tumor site was caused by the accumulation of **D-bpy**, the tissue confocal imaging experiment was carried out, as shown in **Figure S30**, the red fluorescence intensity was much stronger than the green fluorescence intensity of rhodamine derivative, indicating that it mainly exists in the form of **D-bpy**, not the product of the breaking of azo bond. Furthermore, we performed hypoxia-inducible factor (HIF)-1α immunofluorescence staining experiment to test the changes in tumor oxygen levels before and after photodynamic therapy. The results showed that the degree of tumor hypoxia increased after illumination, which was consistent with our expectations (**Figure S31**).

To further evaluate the therapeutic effect of **D-bpy**, **Bpy** and **R-drug**, *in vivo* therapy experiments were implemented. It is worth noting that we used intratumoral injection instead of tail vein injection to compare the therapeutic effects of the three substances to ensure the same dose of the three substances in the tumor area. Two hours after the same dose of **D-bpy**, **Bpy** and **R-drug** were injected intratumorally into the 4T1 tumor-bearing BALB/c mice, two-photon laser (800 nm, 0.8 W/cm^2^) was applied to irradiate the tumor site for 15 min for PDT with deep tissue penetration, and the changes in the tumor volume and body weight were recorded. After two weeks of treatment, the mice were sacrificed and the tumors were excised, weighed and photographed (**Figure 6C, 6D, 6E** and **Figure S32**). It is clear that **D-bpy** could inhibit tumor growth more effectively than **Bpy** and **R-drug** under light conditions. It can be explained that **D-bpy** contains two components, a two-photon photosensitizer, and a hypoxia-activated chemotherapy drug. Upon light irradiation, the photosensitizer can first produce ^1^O_2_ to destroy the cell membrane, thereby promoting more **D-bpy** molecules into the cell freely. The consumption of O_2_ during the photodynamic process exacerbated the degree of hypoxia in the tumor site, triggering a more effective release of the chemotherapeutic drug compared to **R-drug**. However, due to the cell membrane-targeting ability of **D-bpy**, it can remain on the cell membrane without entering the cell in the dark, thereby exhibiting negligible cytotoxicity relative to **R-drug**. Therefore, under light conditions, **D-bpy** has better tumor growth inhibition by combining PDT and chemotherapy and has a much smaller toxicity than **R-drug** in the absence of light. After 16 h of treatment, the mice of each group were also sacrificed, and thereafter, the tumor tissues were excised for histological analysis, as shown in **Figure 6F**. Compared with **Bpy** and **R-drug**, the tumor tissue of the mice injected with **D-bpy** had apparent histological damages after PDT treatment, and had smaller histological damage in the absence of light. The results are in line with our expectations.

The therapeutic effects of **D-bpy** and its effect on the main organs of the organism were also explored through intravenous injection. Firstly, the stability in FBS, PBS and blood of **D-bpy** had been tested (**Figure S33, S34A**), and all results showed that **D-bpy** had good stability. Furthermore, the blood circulation and the biosafety of the **D-bpy** also had be investigated (**Figure S34B, S35**). Most indicators of blood biochemistry and hematology analysis displayed no significant difference from control group. All results showed that **D-bpy** had negligible biological toxicity. Then, **D-bpy** was intravenously injected into the mice. After 6 h, the tumors were irradiated for 15 min with an two-photon laser (800 nm, 0.8 W/cm^2^) for PDT. As shown in **Figure S36A**, the tumor growth in the mice injected with **D-bpy** after irradiation was significantly inhibited, and the tumor volume was nearly 8 times smaller than that of the blank group injected with PBS. After two weeks of treatment, each group of mice was sacrificed, and the tumor tissues were excised, weighed and photographed (**Figure S36B**, **S37**). The result showed no noticeable change in the body-weight of the mice during treatment (**Figure S36C**). Subsequently, the mice with different treatments were sacrificed and the subject organs (spleen, heart, liver, lung and kidneys) were excised for histological analysis. After treatment for 16 h, the mice in each group were sacrificed, and the tumor tissues were excised for histological analysis. As shown in **Figure S36D** and **Figure S38**, only the tumor tissue of the mice treated with **D-bpy** under light irradiation had significant tissue damage, while the main organs (spleen, heart, liver, lung and kidneys) had no apparent histomorphological changes. The above results indicated that intravenous injection of **D-bpy** also has an enhanced therapeutic effect without damage on main organs.

## Conclusions

In summary, a smart-molecule prodrug **D-bpy** was designed and synthesized with cascade activation for programmatic cancer PDT/chemo-therapy. As a multifunctional photosensitizer, **D-bpy** combines two-photon photodynamic therapy and controllable chemotherapy to eliminate deep tumor tissues and achieve good therapeutic effects. Compared with the photosensitizer (**Bpy**) or chemotherapy prodrug (**R-drug**), **D-bpy** has a better therapeutic effect in living mice through the combination of two-photon PDT and chemotherapy. The programmatic treatment strategy in this paper combines two different therapy methods organically, which not only magnifies their advantages, but also avoids their shortcomings. This work provides a new strategy for the design of prodrugs and will shed light on for the development of cancer treatment.

## Supplementary Material

Supplementary figures and tables.

## Abbreviations

PDT: photodynamic therapy
GSH: glutathione
TLC: thin layer chromatography
NMR: nuclear magnetic resonance
H&E: hematoxylin and eosin
OP: one-photon
TP: two-photon
SOSG: singlet oxygen sensor green
DPBF: 1,3-diphenyl-isobenzofuran
MALDI-TOF/MS: matrix-assisted layer desorption/ionization-time-of-flight mass spectrometry
NADPH: nicotinamide adenine dinucleotide phosphate
ROS: reactive oxygen species
DCFH-DA: 2,7-Dichlorofluoresceindiacetate
MTT: 3-(4,5-Dimethyl-2-Thiazolyl)-2,5-Diphenyl Tetrazolium Bromide

## References

[1] Wilson BC, Patterson MS (2008). The physics, biophysics and technology of photodynamic therapy. Phys Med Biol.

[2] Luby BM, Walsh CD, Zheng G (2019). Advanced photosensitizer activation strategies for smarter photodynamic therapy beacons. Angew Chem Int Ed Engl.

[3] Zheng DW, Li B, Li CX, Fan JX, Lei Q, Li C (2016). Carbon-dot-decorated carbon nitride nanoparticles for enhanced photodynamic therapy against hypoxic tumor via water splitting. ACS Nano.

[4] Ouédraogo GD, Redmond RW (2003). Secondary reactive oxygen species extend the range of photosensitization effects in cells: DNA damage produced via initial membrane photosensitization. Photochem Photobiol.

[5] Redmond RW, Kochevar IE (2006). Symposium-in-print: Singlet oxygen invited review. Photochem Photobiol.

[6] Zhang X, Wu FG, Liu P, Gu N, Chen Z (2014). Enhanced fluorescence of gold nanoclusters composed of HAuCl_4_ and histidine by glutathione: Glutathione detection and selective cancer cell imaging. Small.

[7] Yuan Y, Xu S, Zhang CJ, Zhang R, Liu B (2016). Dual-targeted activatable photosensitizers with aggregation-induced emission (AIE) characteristics for image-guided photodynamic cancer cell ablation. J Mater Chem B.

[8] Jiang F, Robin AM, Katakowski M, Tong L, Espiritu M, Singh G (2003). Photodynamic therapy with photofrin in combination with buthionine sulfoximine (BSO) of human glioma in the nude rat. Lasers Med Sci.

[9] Kiesslich T, Plaetzer K, Oberdanner CB, Berlanda J, Obermair FJ, Krammer B (2005). Differential effects of glucose deprivation on the cellular sensitivity towards photodynamic treatment-based production of reactive oxygen species and apoptosis-induction. FEBS Lett.

[10] Henderson BW, Miller AC (1986). Effects of scavengers of reactive oxygen and radical species on cell survival following photodynamic treatment *in vitro*: comparison to ionizing radiation. Radiat Res.

[11] Hall MD, Hambley TW (2002). Platinum (IV) antitumour compounds: their bioinorganic chemistry. Coord Chem Rev.

[12] Liang H, Zhou Z, Luo R, Sang M, Liu B, Sun M (2018). Tumor-specific activated photodynamic therapy with an oxidation-regulated strategy for enhancing anti-tumor efficacy. Theranostics.

[13] Fan H, Yan G, Zhao Z, Hu X, Zhang W, Liu H (2016). A smart photosensitizer-manganese dioxide nanosystem for enhanced photodynamic therapy by reducing glutathione levels in cancer cells. Angew Chem Int Ed Engl.

[14] Ju E, Dong K, Chen Z, Liu Z, Liu C, Huang Y (2016). Copper(II)-graphitic carbon nitride triggered synergy: Improved ROS generation and reduced glutathione levels for enhanced photodynamic therapy. Angew Chem Int Ed Engl.

[15] Wang C, Cao F, Ruan Y, Jia X, Zhen W, Jiang X (2019). Specific generation of singlet oxygen through the russell mechanism in hypoxic tumors and GSH depletion by CU-TCPP nanosheets for cancer therapy. Angew Chem Int Ed Engl.

[16] Liu Y, Zhou Z, Liu Y, Li Y, Huang X, Qian C (2019). H_2_O_2_-activated oxidative stress amplifier capable of gsh scavenging for enhancing tumor photodynamic therapy. Biomater Sci.

[17] Wang D, Su H, Kwok RTK, Hu X, Zou H, Luo Q (2018). Rational design of a water-soluble NIR AIEgen, and its application in ultrafast wash-free cellular imaging and photodynamic cancer cell ablation. Chem Sci.

[18] Draeger A, Monastyrskaya K, Babiychuk EB (2011). Plasma membrane repair and cellular damage control: the annexin survival kit. Biochem Pharmacol.

[19] Chapman D (1974). Biological membranes. Thromb Res.

[20] Tate SS, Meister GA (1979). Conversion of glutathione to glutathione disulfide by cell membrane-bound oxidase activity. Proc Natl Acad Sci U S A.

[21] Brown SB, Brown EA, Walker I (2004). The present and future role of photodynamic therapy in cancer treatment. Lancet Oncol.

[22] Nonaka Y, Nanashima A, Nonaka T, Uehara M, Isomoto H, Abo T (2013). Synergic effect of photodynamic therapy using talaporfin sodium with conventional anticancer chemotherapy for the treatment of bile duct carcinoma. J Surg Res.

[23] Diez B, Ernst G, Teijo MJ, Batlle A, Hajos S, Fukuda H (2012). Combined chemotherapy and ala-based photodynamic therapy in leukemic murine cells. Leuk Res.

[24] Wan H, Zhang Y, Zhang W, Zou H (2015). Robust two-photon visualized nanocarrier with dual targeting ability for controlled chemo-photodynamic synergistic treatment of cancer. ACS Appl Mater Interfaces.

[25] Wang T, Zhang L, Su Z, Wang C, Liao Y, Fu Q (2011). Multifunctional hollow mesoporous silica nanocages for cancer cell detection and the combined chemotherapy and photodynamic therapy. ACS Appl Mater Interfaces.

[26] Pei Q, Hu X, Zheng X, Liu S, Li Y, Jing X (2018). Light-activatable red blood cell membrane-camouflaged dimeric prodrug nanoparticles for synergistic photodynamic/chemotherapy. ACS Nano.

[27] Li XS, Lee SY, Yoon J (2018). Supramolecular photosensitizers rejuvenate photodynamic therapy. Chem Soc Rev.

[28] Li XS, Yu S, Lee D, Kim G, Lee B, Cho Y (2018). Facile supramolecular approach to nucleicacid-driven activatable nanotheranostics that overcome drawbacks of photodynamic therapy. ACS Nano.

[29] Li XS, Lovell JF, Yoon J, Chen XY (2020). Clinical development and potential of photothermal and photodynamic therapies for cancer. Nat Rev Clin Oncol.

[30] Liu LH, Qiu WX, Li B, Zhang C, Sun LF, Wan SS (2016). A red light activatable multifunctional prodrug for image-guided photodynamic therapy and cascaded chemotherapy. Adv Funct Mater.

[31] Elliott CM, Hershenhart EJ (1982). Electrochemical and spectral investigations of ring-substituted bipyridine complexes of ruthenium. J Am Chem Soc.

[32] Yao HW, Zhu XY, Guo XF, Wang H (2016). An amphiphilic fluorescent probe designed for extracellular visualization of nitric oxide released from living cells. Anal Chem.

[33] Lee MH, Jeon HM, Han JH, Park N, Kang C, Sessler JL (2014). Toward a chemical marker for inflammatory disease: a fluorescent probe for membrane-localized thioredoxin. J Am Chem Soc.

[34] Xu S, Liu HW, Yin X, Yuan L, Huan SY, Zhang XB (2019). A cell membrane-anchored fluorescent probe for monitoring carbon monoxide release from living cells. Chem Sci.

[35] Shi L, Li K, Liu Y-H, Liu X, Zhou Q, Xu Q (2020). Bio-inspired assembly in a phospholipid bilayer: effective regulation of electrostatic and hydrophobic interactions for plasma membrane specific probes. Chem Commun.

[36] Heinemann F, Karges J, Gasser G (2017). Critical overview of the use of Ru(II) polypyridyl complexes as photosensitizers in one-photon and two-photon photodynamic therapy. Acc Chem Res.

[37] Liu J, Zhang C, Rees TW, Ke L, Ji L, Chao H (2018). Harnessing ruthenium(II) as photodynamic agents: encouraging advances in cancer therapy. Coord Chem Rev.

[38] Zhou Z, Liu J, Rees TW, Wang H, Li X, Chao H (2018). Heterometallic Ru-Pt metallacycle for two-photon photodynamic therapy. Proc Natl Acad U S A.

[39] Ai XZ, Mu J, Xing BG (2016). Recent advances of light-mediated theranostics. Theranostics.

[40] Bi XD, Yang R, Zhou YC, Chen DM, Li GK, Guo YX (2020). Cyclometalated iridium(III) complexes as high-sensitivity two-photon excited mitochondria dyes and near-infrared photodynamic therapy agents. Inorg Chem.

[41] Liu JP, Liao XX, Xiong K, Kuang S, Jin CZ, Ji LN (2020). Boosting two-photon photodynamic therapy with mitochondria-targeting ruthenium-glucose conjugates. Chem Commun.

[42] Juvekar V, Lim CS, Lee DJ, Park SJ, Song GO, Kang H (2021). An azo dye for photodynamic therapy that is activated selectively by two-photon excitation. Chem Sci.

[43] Huang H, Yu B, Zhang P, Huang J, Chen Y, Gasser G (2015). Highly charged ruthenium(II) polypyridyl complexes as lysosome-localized photosensitizers for two-photon photodynamic therapy. Angew Chem Int Ed Engl.

[44] Mari C, Pierroz V, Ferrari S, Gasser G (2015). Combination of Ru(ii) complexes and light: new frontiers in cancer therapy. Chem Sci.

[45] Knoll JD, Turro C (2015). Control and utilization of ruthenium and rhodium metal complex excited states for photoactivated cancer therapy. Coord Chem Rev.

[46] Joshi T, Pierroz V, Mari C, Gemperle L, Ferrari S, Gasser G (2014). A bis(dipyridophenazine)(2-(2-pyridyl)pyrimidine-4-carboxylic acid)ruthenium(II) complex with anticancer action upon photodeprotection. Angew Chem Int Ed Engl.

[47] Verwilst P, Han J, Lee J, Mun S, Kang H-G, Kim JS (2017). Reconsidering azobenzene as a component of small-molecule hypoxia-mediated cancer drugs: a theranostic case study. Biomaterials.

[48] Kondo N, Takahashi A, Ono K, Ohnishi T (2010). DNA damage induced by alkylating agents and repair pathways. J Nucleic Acids.

[49] Loeber RL, Michaelson-Richie Ed, Codreanu SG, Liebler DC, Campbell CR, Tretyakova NY (2009). Proteomic analysis of DNA-protein cross-linking by antitumor nitrogen mustards. Chem Res Toxicol.

[50] Hay MP, Anderson RF, Ferry DM, Wilson WR, Denny WA (2003). Synthesis and evaluation of nitroheterocyclic carbamate prodrugs for use with nitroreductase-mediated gene-directed enzyme prodrug therapy. J Med Chem.

[51] Hu M, Yang C, Luo Y, Chen F, Yang F, Yang S (2018). A hypoxia-specific and mitochondria-targeted anticancer theranostic agent with high selectivity for cancer cells. J Mater Chem B.

[52] Ding N, Li Z, Tian X, Zhang J, Guo K, Wang P (2019). Azo-based near-infrared fluorescent theranostic probe for tracking hypoxia-activated cancer chemotherapy *in vivo*. Chem Commun.

[53] Shen J, Xue T, He Y (2020). An enzyme-activable noncovalent fluorescent probe based on water soluble azobenzene containing polymer and AIEgen. Macromol Chem Phys.

[54] Lakowicz JR, Bevan DR, Maliwal BP, Cherek H, Balter A (1983). Synthesis and characterization of a fluorescence probe of the transition and dynamic properties of membranes. Biochemistry.

[55] Li M, Xiong T, Du J, Tian R, Xiao M, Guo L (2019). Superoxide radical photogenerator with amplification effect: Surmounting the achilles' heels of photodynamic oncotherapy. J Am Chem Soc.

[56] Chevalier A, Mercier C, Saurel L, Orenga S, Renard PY, Romieu A (2013). The first latent green fluorophores for the detection of azoreductase activity in bacterial cultures. Chem Commun.

[57] Chen B, Le W, Wang Y, Li Z, Wang D, Lin L (2016). Targeting negative surface charges of cancer cells by multifunctional nanoprobes. Theranostics.

