# Supplementary material for: Cell membranes targeted unimolecular prodrug for programmatic photodynamic-chemo therapy
Source: Theranostics. 2021 Jan 19;11(7):3502–11. doi: 10.7150/thno.55014 (PMC7847693; doi:10.7150/thno.55014)
Supplement: Supplementary file 1 — Supplementary figures and tables. [file thnov11p3502s1.pdf]

Supporting Information for

Cell membranes targeted unimolecular prodrug for programmatic photodynamic-chemo therapy

Jie Yuan[1], Rong Peng[1], Dongdong Su[2], Xingxing Zhang[1], Hepeng Zhao[3], Xiujuan Zhuang[3], Mei Chen[4], Xiaobing Zhang[1], Lin Yuan[1]*

1. State Key Laboratory of Chemo/Biosensing and Chemometrics, College of Chemistry and Chemical Engineering, Hunan University, Changsha 410082, P. R China.

2. Department of Chemistry and Chemical Engineering, Beijing University of Technology, Beijing, 100124, P. R. China

3. College of Physics and Microelectronics Science, Hunan University, Changsha 410082, P. R China

4. College of Materials Science and Engineering, Hunan University, Changsha 410082, P. R China

*Corresponding author: lyuan@hnu.edu.cn

Experiment Section

Synthesis and Characterization

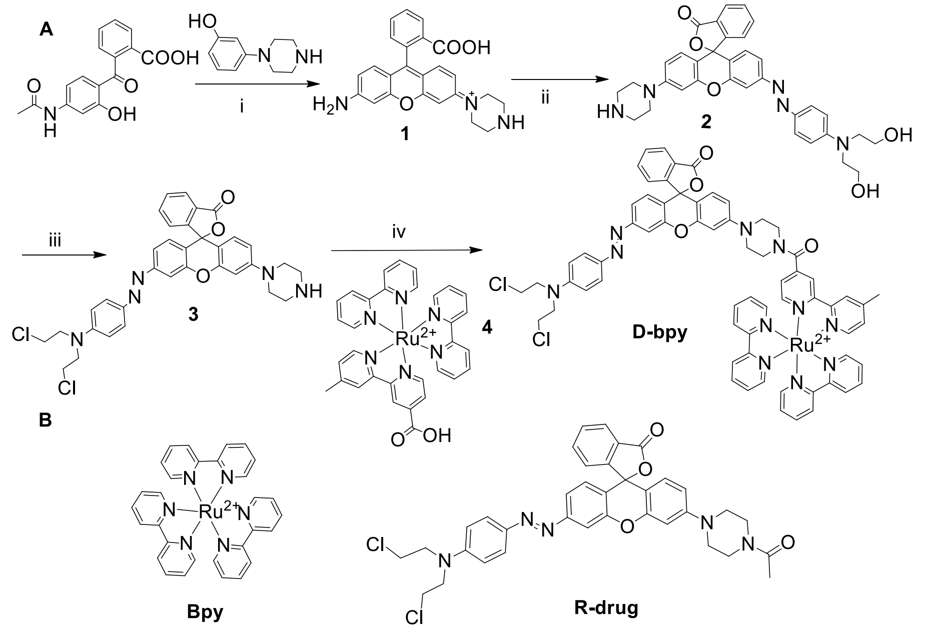

Scheme S1. Structure and synthetic route. (A) Synthesis of **D-bpy**. (i) $MeSO_3H$, 100 ºC. (ii) $NaNO_2$, sulfamic acid, N-Phenyldiethanolamine, $MeCN/CH_2Cl_2$, 1 % TFA, N_2, 0 ºC. (iii) DCM, $SOCl_2$, reflux. (iv) $MeCN/CH_2Cl_2$, HATU, 25 ºC. (B) Structures of **Bpy** and **R-drug**.

Compound 2: Compound **1** [1, 2] (280.0 mg, 0.38 mmol) was firstly dissolved in $MeCN/CH_2Cl_2$ (1: 4, 16 mL) containing 1% TFA, and then the solution was stirred at 0 ℃ under N_2 atmosphere. Then $NaNO_2$ (59.0 mg, 0.76 mmol) was added into the above mixture and continued stirring at 0 ℃ for 10 min. Sulfamic acid (74.0 mg, 0.76 mmol) was then added into the mixture and then continued stirring for 5 min. Next, the N-Phenyldiethanolamine (414.1 mg, 2.28 mmol) in MeCN (2 mL) was added. Then the above mixture was stirred at 0 ℃ for 2 h, diluted with H_2O and then extracted with CH_2Cl_2. The organic layer was dried using anhydrous Na_2SO_4 and then the solvent was evaporated to dryness. The crude material was further purified by chromatography column (silica gel, DCM→DCM/MeOH 100/3) to obtain a deep yellow solid (Yield 65%). ^1H NMR (400 MHz, $CDCl_3$) δ 8.05 (d, J = 7.3 Hz, 1H), 7.87 (d, J = 8.1 Hz, 2H), 7.72 (s, 1H), 7.71 – 7.61 (m, 2H), 7.51 (d, J = 8.4 Hz, 1H), 7.18 (d, J = 7.3 Hz, 1H), 6.89 (d, J = 8.4 Hz, 1H), 6.75 (dd, J = 15.2, 8.3 Hz, 4H), 6.64 (d, J = 8.8 Hz, 1H), 4.47 – 4.38 (m, 2H), 3.95 (dd, J = 10.9, 7.1 Hz, 6H), 3.72 (s, 4H),

3.55 – 3.50 (m, 2H), 3.30 (t, J = 5.0 Hz, 2H). ^{13}C NMR (101 MHz, $CDCl_3$) δ 169.5, 154.5, 153.2, 152.5, 151.9, 151.8, 150.8, 143.9, 135.1, 129.8, 129.0, 128.5, 126.4, 125.4, 125.1, 123.9, 119.5, 117.9, 112.6, 112.1, 110.3, 110.2, 103.1, 82.8, 60.8, 55.2, 49.2, 48.8, 47.3, 39.4. MALDI-TOF/MS (*m/z*): Calcd for $[C_{34}H_{33}N_5O_5+H]^+$: 592.24, Found: 592.42.

Compound 3: Compound **2** (100.1 mg, 0.17 mmol) was dissolved in 20 mL dry DCM, then 0.5 mL $SOCl_2$ was added into it and the resulting solution was heated to 44 ℃ for 2 h. After the solution cooling to room temperature, th e mixture was evaporated to dryness. After a chromatographic separation (silica gel, DCM→DCM/MeOH 100/3) the final target compound was obtained (Yield 72%).^{1}H NMR (400 MHz, $CDCl_3$) δ 7.97 (d, J = 6.9 Hz, 1H), 7.82 (d, J = 8.1 Hz, 2H), 7.66 – 7.52 (m, 3H), 7.44 (d, J = 8.6 Hz, 1H), 7.10 (d, J = 7.1 Hz, 1H), 6.82 (d, J = 8.3 Hz, 1H), 6.69 (d, J = 7.7 Hz, 3H), 6.63 (d, J = 8.5 Hz, 1H), 6.54 (d, J = 8.6 Hz, 1H), 3.76 (d, J = 6.2 Hz, 4H), 3.61 (dd, J = 13.4, 7.2 Hz, 4H), 3.57 (s, 1H), 3.38 (s, 3H), 3.19 (s, 4H). ^{13}C NMR (101 MHz, $CDCl_3$) δ 169.8, 154.9, 153.6, 152.8, 152.2, 152.1, 151.2, 144.2, 135.4, 130.1, 129.4, 128.9, 126.8, 125.8, 125.5, 124.2, 119.9, 118.3, 112.9, 112.4, 110.6, 110.5, 103.4, 83.1, 61.0, 55.5, 49.5, 49.2, 47.6, 39.8. MALDI-TOF/MS (*m/z*): Calcd for $[C_{34}H_{31}Cl_2N_5O_3+H]^+$: 628.18, Found: 628.36.

Compound D-bpy: Compound **4** [3, 4] (76.0 mg, 0.12 mmol) was dissolved in CH_3CN/CH_2Cl_2 = 2/1 (6 mL), then 1-[bis(dimethylamino)methylene]-1H-1,2,3-triazolo[4,5-b]pyridinium 3-oxide hexafluorophosphate (HATU, 90.0 mg, 0.24 mmol) was added into it. Then the above solution was stirred at room temperature for 5 min and compound **3** (80.0 mg, 0.13 mmol) was added into it. After stirring at room temperature overnight, the solvent was evaporated to dryness to obtain a dark green solid. After a chromatographic separation (silica gel, $KNO_3/H_2O/CH_3CN$ = 1/1/7) to obtain **D-bpy** as a dark green solid (Yield 15%). ^{1}H NMR (400 MHz, CD_3CN) δ 8.55 (d, J = 8.1 Hz, 4H), 8.50 (s, 1H), 8.47 (s, 1H), 8.14 – 8.04 (m, 5H), 7.89 (d, J = 8.6 Hz, 3H), 7.86 – 7.72 (m, 7H), 7.60 (d, J = 5.8 Hz, 1H), 7.54 (dd, J = 8.5, 1.6 Hz, 1H), 7.50 – 7.38 (m, 5H), 7.30 (dd, J = 10.9, 6.6 Hz, 2H), 6.97 (dd, J = 12.7, 8.8 Hz, 3H), 6.86 (s, 1H), 6.76 (d, J = 7.9 Hz, 2H), 3.90 (t, J = 6.7 Hz, 6H), 3.80 (t, J = 6.8 Hz, 4H), 3.58 (s, 2H), 3.45 (s, 2H), 3.32 (s, 2H), 2.57 (s, 3H). ^{13}C NMR (101 MHz, CD_3CN) δ 168.9, 165.4, 157.5, 156.7, 156.6,

156.6, 155.7, 154.1, 152.6, 152.5, 152.0, 151.9, 151.6, 151.5, 151.4, 151.2, 150.5, 150.3, 149.8, 144.2, 143.6, 137.5, 135.2, 129.8, 128.6, 128.5, 128.4, 127.3, 127.3, 126.0, 125.2, 125.0, 124.5, 123.9, 123.6, 121.5, 119.8, 112.1, 111.8, 109.8, 108.6, 101.6, 52.2, 40.3, 29.0, 19.9. MALDI-TOF/MS (*m/z*): Calcd for $[C_{66}H_{55}Cl_2N_{13}O_{10}Ru\text{-}2NO_3]^+$: 1238.285, Found: 1238.600. And after a chromatographic separation (silica gel, $KNO_3/H_2O/CH_3CN$ = 1/1/7), the resulting solution was also evaporated to 3 mL and treated with NH_4PF_6 to precipitate the product for ESI-QTOF-MS: *m/z* = 1384.27 $[M\text{-}PF_6\text{+}H]^+$(calcd: 1384.27), 619.63 $[M\text{-}2PF_6\text{+}H]^+$ (calcd: 619.65).

Spectrometric Studies.

For photophysical characterization, the compound D-bpy was dissolved in DMSO to make the stock solutions (500 μM), which was diluted to 5 μM as the testing solutions with PBS buffer solution. Absorption and fluorescence spectroscopic studies were performed on a UV 1800 ultraviolet and visible spectrophotometer and a Hitachi F-4600 fluorescence spectrophotometer, respectively.

Measurement of Two-Photon Cross Section. The two-photon cross section (σ) was determined by a femtosecond (fs) fluorescence measurement technique. **D-bpy** was dissolved in MeOH, at a concentration of 5.0 × 10^{-6} M, and then the two-photon fluorescence intensity was measured at 750-850 nm by using rhodamine B in MeOH as the standard, whose two-photon properties have been well-characterized in the literature [5]. The two-photon cross-section was calculated from equations:

$$\sigma = \sigma_r (F_s n_s^2 \Phi_r \ C_r)/ (F_r n_r^2 \Phi_s C_s)$$

where the subscripts s and r stand for sample and reference molecules, F is the average fluorescence intensity, n is the refractive index of the solvent, C is the concentration, Φ is the quantum yield, and σ_r is the two-photon cross-section of the reference molecule.

$^1O_2$ Generation of D-bpy in Solution

The $^1O_2$ quantum yields were measured by monitoring the photooxidation of DPBF (40 μM) in methanol in the presence of **D-bpy**. The OD460 nm values of **D-bpy** and $[Ru(bpy)_3]^{2+}$(**Bpy**) solutions were 0.135 and 0.131, respectively. The methanol solution with **D-bpy**/**Bpy** /methanol (control) and DPBF were fully aerated and were subjected LED irradiation (450-470 nm). The absorbance of the mixture at 411 nm was recorded every 20 s. **Bpy** was used as the reference in methanol (Φ = 81%). The quantum yield of **D-bpy** was calculated by the following equations:

$$\emptyset_{\triangle}^{s} = \emptyset_{\triangle}^{r} \times (S^s \times F^r)/(S^r \times F^s)$$

$$F = 1\text{-}10^{-OD\ 460nm}$$

where *s* denotes the calibrated slope of a linear fit of the cumulative changes of absorbance at 411 nm *vs.* cumulative irradiation time. F represents the absorption

correction factor. Superscript "*s*" stands for the sample, and "*r*" stands for the reference, i.e. $[Ru(bpy)_3]^{2+}$(**Bpy**).

Enzyme Reduction Reaction. The 0.1 M potassium phosphate buffer (pH 7.4) was bubbled with Ar for 2 h to remove O_2 remaining in the solution. Then **D-bpy** (10 μM), 50 μL rat liver microsomes, and NADPH (50 μM) were dissolved in potassium phosphate buffer (pH 7.4) and reacted at 37 ℃ for 12 h under Ar atmosphere.

Single photon and two-photon induced cellular singlet oxygen generation

The intracellular ROS under OP and TP irradiation was measured using the fluorescent probe 2′,7′-Dichlorofluorescein diacetate (**DCFH-DA**). **DCFH-DA** diffuses through the cell membrane and is enzymatically hydrolyzed by intracellular esterases to form the non-fluorescent compound **DCFH**, which is then rapidly oxidized to form the highly fluorescent 2′,7′-Dichlorofluorescein (**DCF**) in the presence of ROS. The **DCF** fluorescence intensity is believed to parallel the amount of ROS formed intracellularly. The cultured cancerous cells were treated with 5 μM of **D-bpy** in the dark. After 1 h the cells were incubated with 10 μM of **DCFH-DA** at 37 °C for 10 min, the cells were subjected to OP (488 nm) and TP (800 nm) irradiation, respectively. The excitation wavelength of intracellular ROS was set as 488 nm, and the fluorescence was measured at 500-550 nm.

The intracellular $^1O_2$ generation of **D-bpy** under light irradiation was also detected using the specific singlet oxygen indicator SOSG. The experimental procedure is the same as above.

Fluorescence Imaging of Hypoxia in HeLa Cells by D-bpy.

HeLa cell were grown on glass-bottom culture dishes at 37 °C under normoxic (21% O_2) and hypoxic (1% O_2). In our experiments, these cells were incubated with **D-bpy** (5 μM) under normoxic and hypoxic conditions at 37 ℃ for 6 h, and then to observe the fluorescence imaging with a fluorescence microscope. The fluorescence emission signals from rhodamine derivative (500-550 nm) were collected by irradiation with 488 nm light.

Octanol/water partition coefficient (log Po/w)

The partition-coefficient of each complex, expressed as

$$\log P^{0}_{o/w} = \log \frac{[solute]_{octanol}}{[solute]_{water}}$$

was determined by "shake-flask" method with a little modified. Water and octanol were mixed and shook thoroughly to reach equilibrium, which results in separation of two layers, i.e., water saturated with octanol and octanol saturated with water. The two layers were separated for following experiment. The **D-bpy** and **Bpy** were dissolved using MeOH firstly to obtain a standard solution (500 μM), using to draw the standard curve. The standard solution were then used to obtain the sample liquid by adding octanol saturated with water. Taking a certain volume of sample solution and nine volume of water phase previously saturated with octanol was then added to the solution. The mixture was shaken at room temperature for 24 h. The concentration of **D-bpy** and **Bpy** were determined by UV-vis spectroscopy using the extinction coefficients of the complexes in water saturated with octanol. The evaluation was replicated three times.

Cell cytotoxicity in MTT assay

The HeLa cells were seeded in 96 well cell culture plate at density of 10000 cells per well for 24 h. **D-bpy**, **Bpy** and **R-drug** at different concentrations were added into each well and cultured for 4 h. The cell medium was replaced with fresh medium and then the cells were irradiated upon 450-470 nm LED light. After 24 h of treatment, the MTT (0.5 mg/mL) reagent was added for 4 hours at 37 ℃ and DMSO (100 μL/well) was further incubated with cells to dissolve the precipitated formazan violet crystals at 37 ℃ for 15 min. The absorbance was measured at 490 nm by a multidetection microplate reader. The following formula was used to calculate the viability of cell growth: Cell viability (%) = (mean of A value of treatment group / mean of A value of control) × 100.

For dark toxicity of **D-bpy**, **Bpy** and **R-drug**, no light irradiation was applied to this experiment, and all other steps were the same.

We also used 4T1 cells performed the same cell cytotoxicity experiment as HeLa cells.

Dead/Live Cell Co-staining.

Firstly, HeLa cells were incubated on the cell culture plate for 24 h, then exposed to different following treatments: group one, untreated (Control); group two, incubated with **D-bpy** (5 μM) at 37 ℃ for 1 h; group three, incubated with **D-bpy** (5 μM) for 1 h at 37 ℃ and irradiated with 450-470 nm LED light for 20 min under normoxia, (21% O_2, **D-bpy** + light). After different treatments, Calcein AM and Propidium Iodide co-staining was performed for visualization of dead apoptotic cells.

Next, we performed the live cell/dead cell staining experiments to confirm the synergistic effect. Firstly, 4T1 cells were incubated on the cell culture plate for 24 h, then exposed to different following treatments: group one, incubated under 1% O_2 conditions at 37 ℃ for 6 h (Control); group two, incubated with **R-drug** (5 μM) under 1% O_2 conditions at 37 ℃ for 6 h; group three, incubated with **D-bpy** (5 μM) under 1% O_2 conditions at 37 ℃ for 6 h and irradiated with 450-470 nm LED light for 8 min. group four, incubated with **Bpy** (5 μM) under 1% O_2 conditions at 37 ℃ for 6 h and irradiated with 450-470 nm LED light for 8 min. After different treatments, Calcein AM and Propidium Iodide co-staining was performed for visualization of dead apoptotic cells.

Hypoxia-inducible factor (HIF-1α) immune fluorescence staining experiment

The hypoxia was evaluated via hypoxia-inducible factor (HIF-1α) immunofluorescence staining. Tumor-bearing mice were randomly divided into four groups. The **D-bpy** was injected intravenously in all groups. After 6 h, the group one, group two and group three were irradiated with light for PDT while the group four (control) was not.

The stability of D-bpy in FBS and PBS

For the stability test, the same amount of **D-bpy** with FBS and PBS were incubated for different times, respectively. Then the fluorescence intensity at 550 nm (rhodamine derivative) and 644 nm (**D-bpy**) at different times were tested, respectively.

The blood stability and blood circulation and biosafety of D-bpy

For the blood stability test, the same amount of **D-bpy** with blood were incubated for different times, and fluorescence intensity at 550 nm (rhodamine derivative) and 644 nm (**D-bpy**) at different times were tested, respectively.

For pharmacokinetic study, healthy Balb/c mice (n = 3) received intravenous injection with **D-bpy** (200 μL, 200 μM). At indicated time points, the blood was withdrawn, the ruthenium content in the blood was quantified by ICP-MS measurements for metabolism analysis. Untreated mice were used as the reference.

For blood biochemistry and hematology analysis, For pharmacokinetic study, healthy Balb/c mice (n = 3) received intravenous injection with **D-bpy** (100 μL, 500 μM). Then the blood samples were collected using the standard protocol, and then sent to Wuhan Service bio Technology Co., Ltd. For blood analysis.

Figures

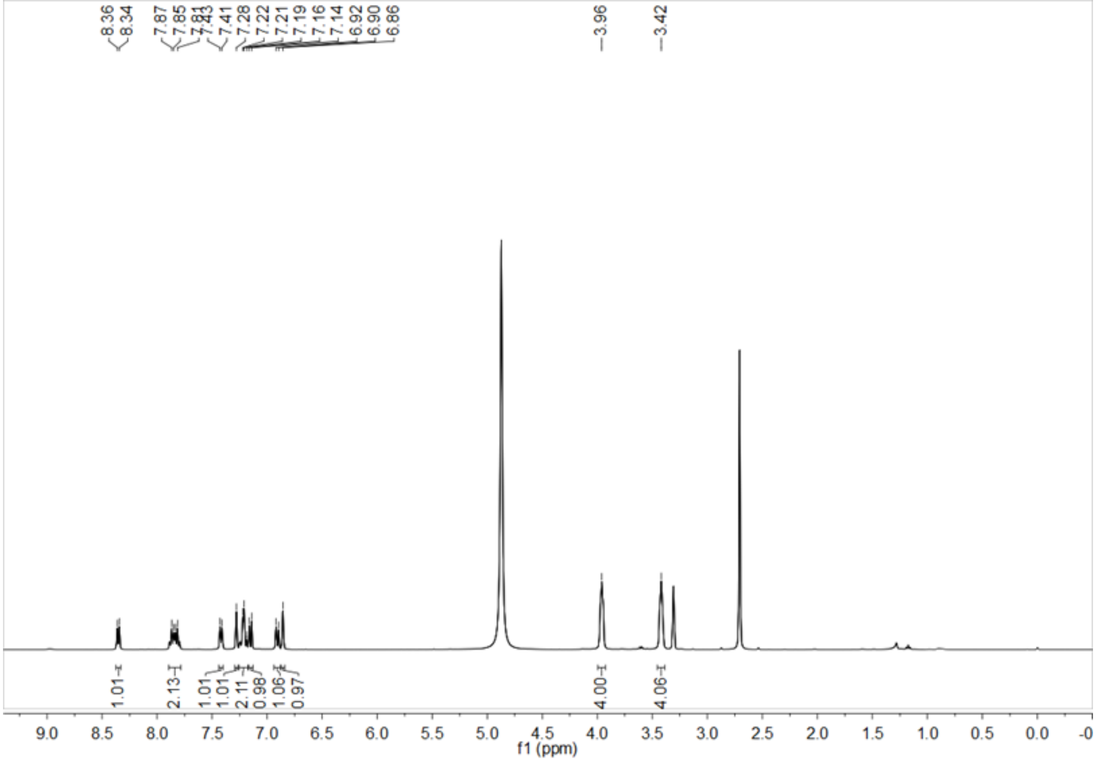

Figure S1 The ^{1}HNMR spectrum of compound **1** (CD_3OD).

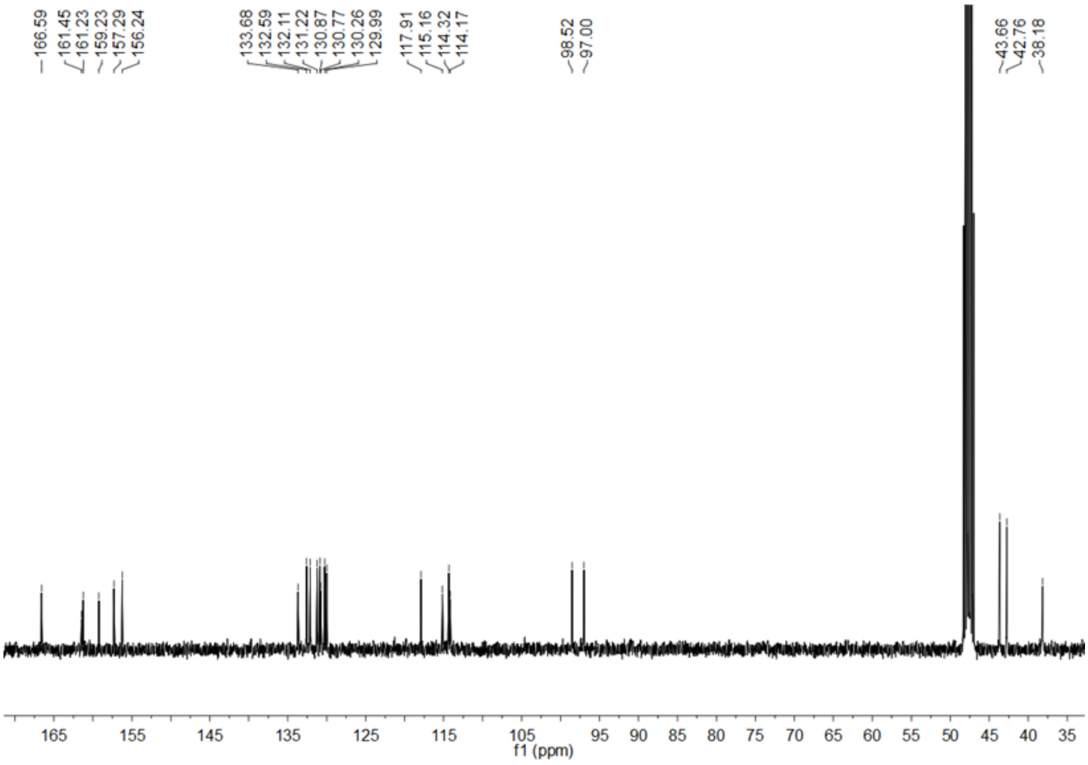

Figure S2 The ^{13}CNMR spectrum of compound **1** (CD_3OD).

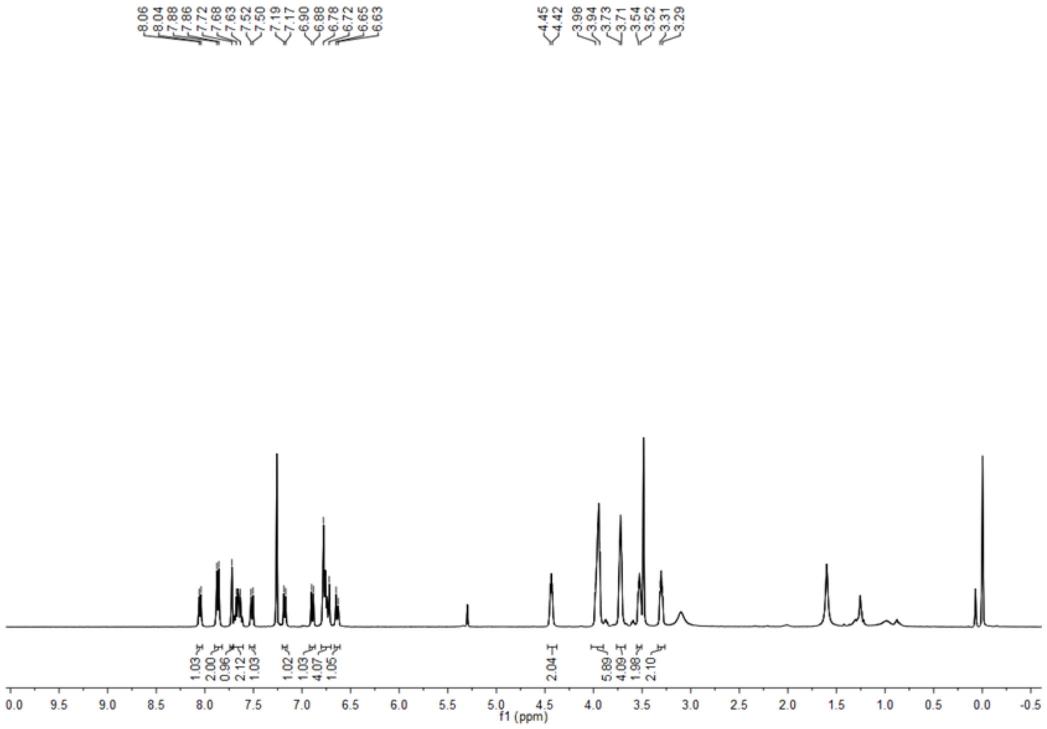

Figure S3 The ^{1}HNMR spectrum of compound **2**(CDCl$_3$)**.**

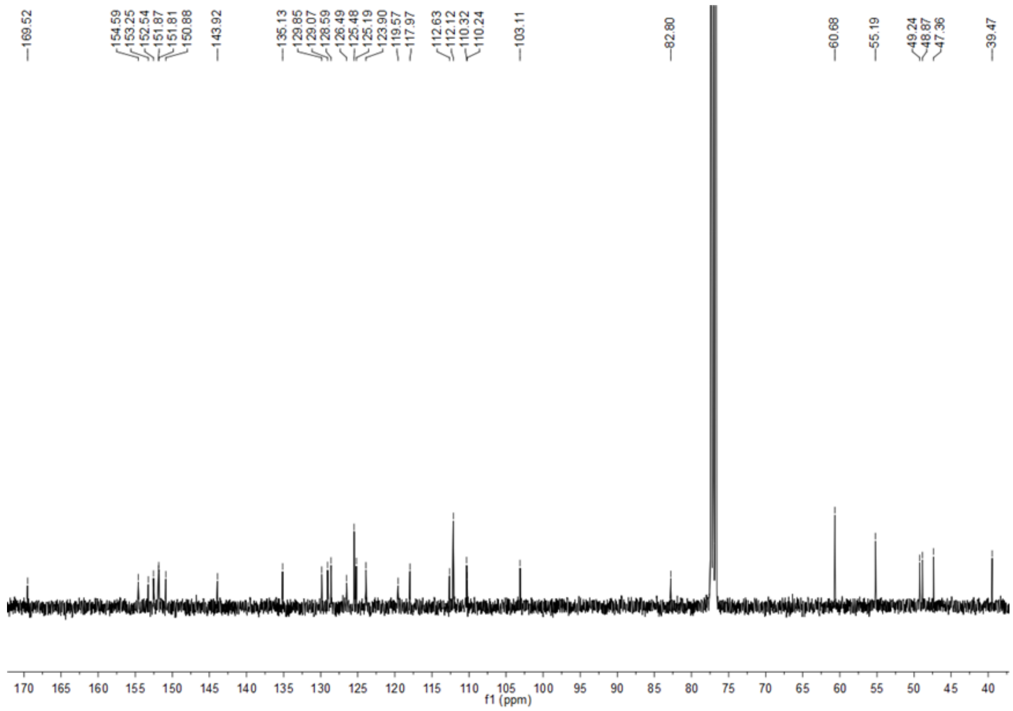

Figure S4 The ^{13}CNMR spectrum of compound **2 (**CDCl$_3$)**.**

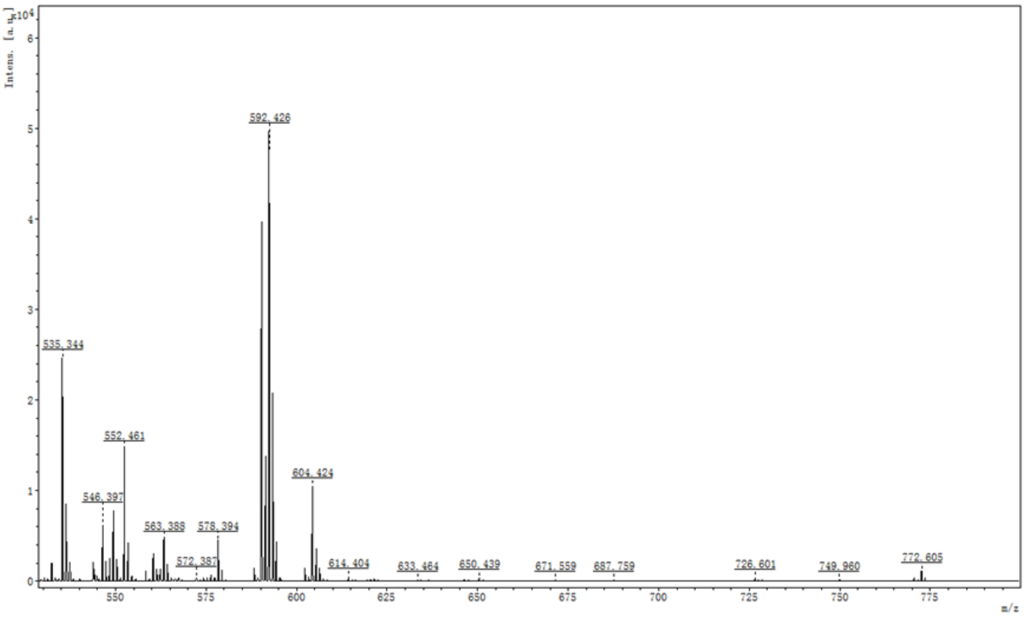

Figure S5 MALDI-TOF/MS spectrum of compound **2.**

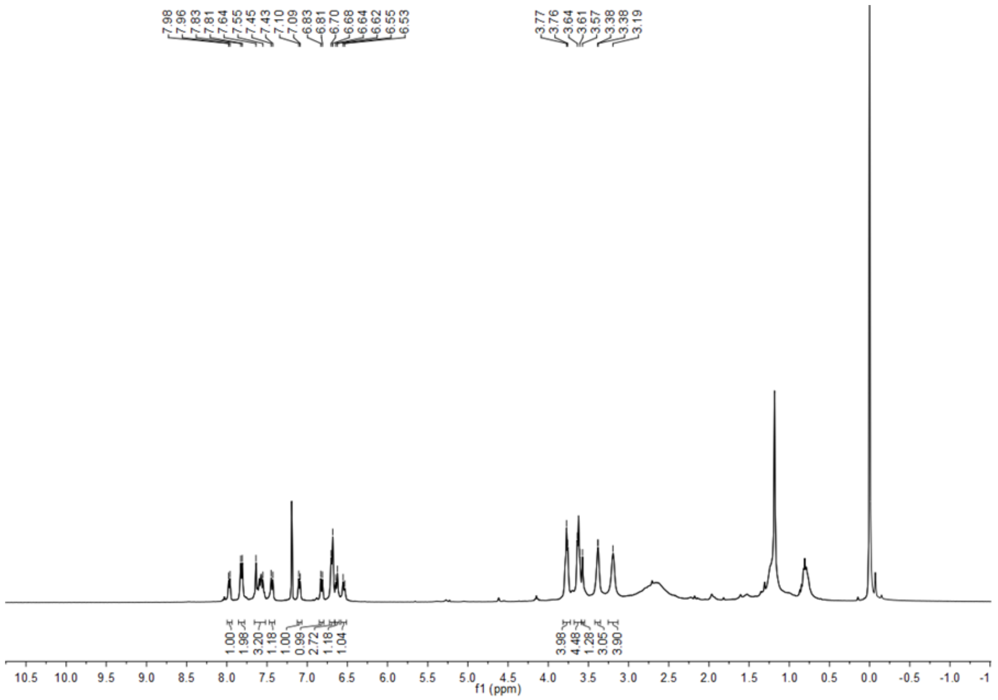

Figure S6 The ^{1}HNMR spectrum of compound **3** ($CDCl_3$)**.**

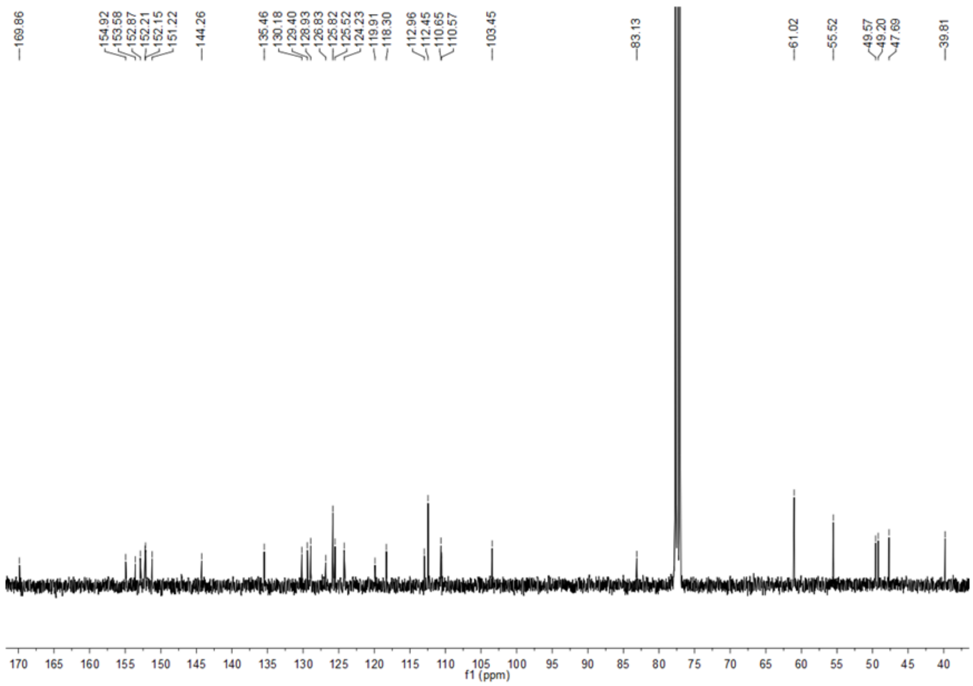

Figure S7 The ^{13}CNMR spectrum of compound **3** ($CDCl_3$)**.**

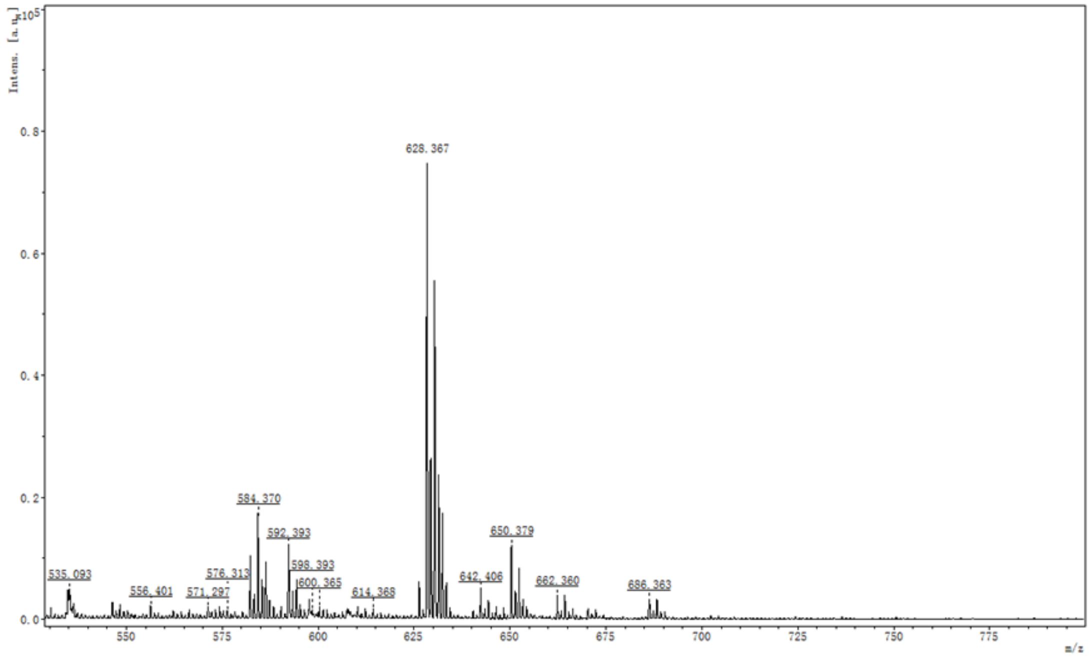

Figure S8 MALDI-TOF/MS spectrum of compound **3.**

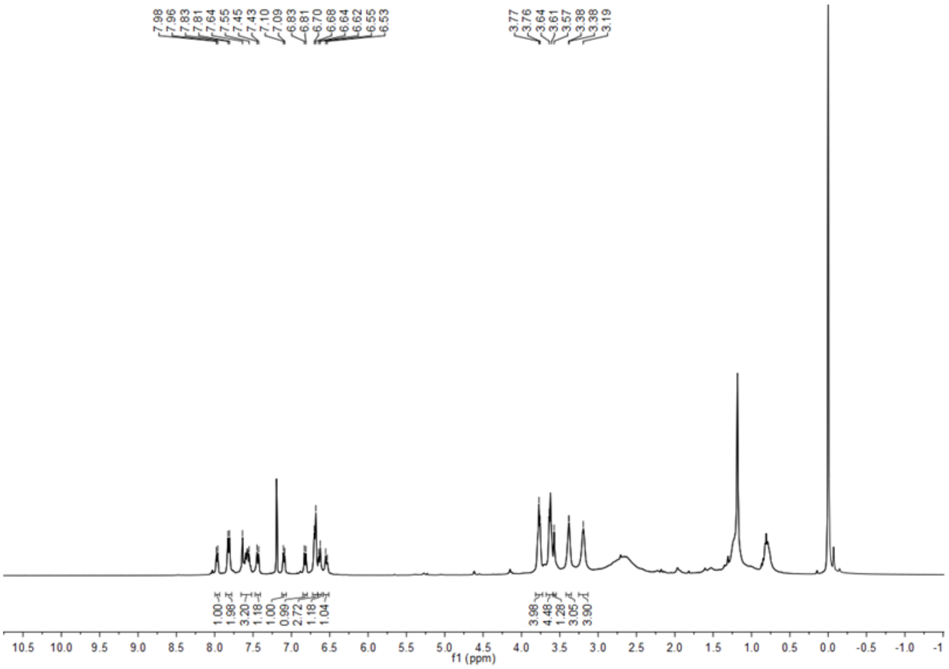

Figure S9 The ^{1}HNMR spectrum of compound **D-bpy** (CD_3CN)**.**

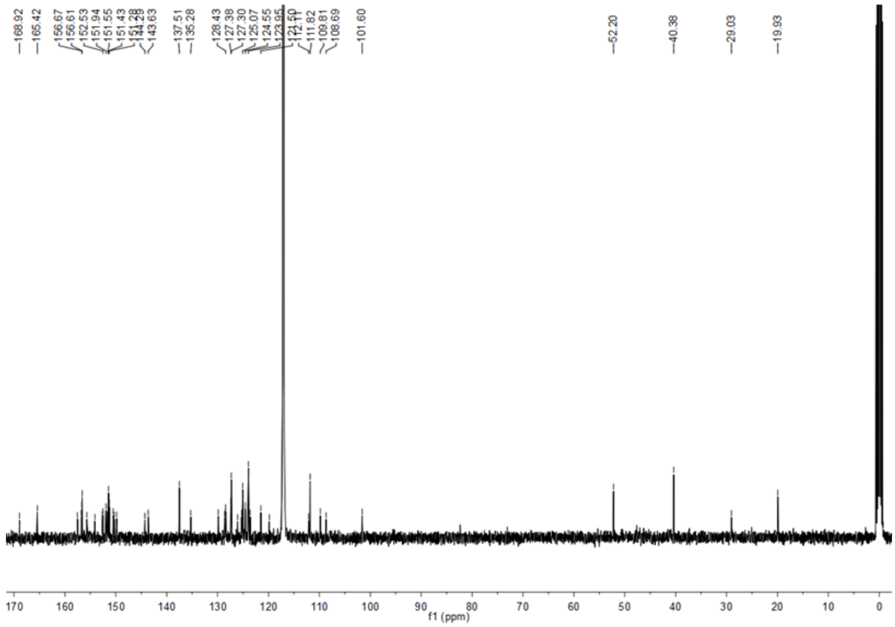

Figure S10 The ^{13}CNMR spectrum of compound **D-bpy** (CD_3CN).

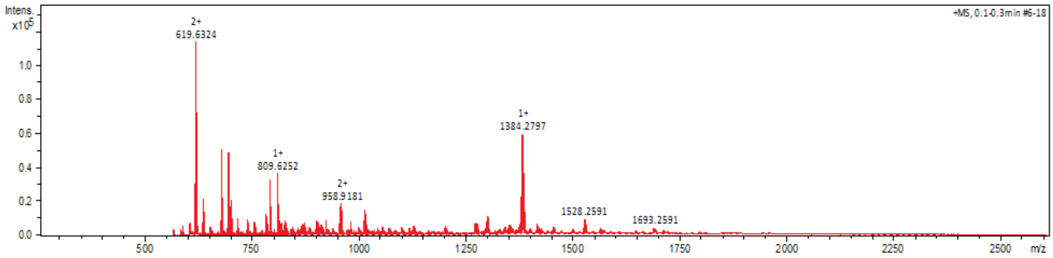

Figure S11 ESI-QTOF-MS of **D-bpy**.

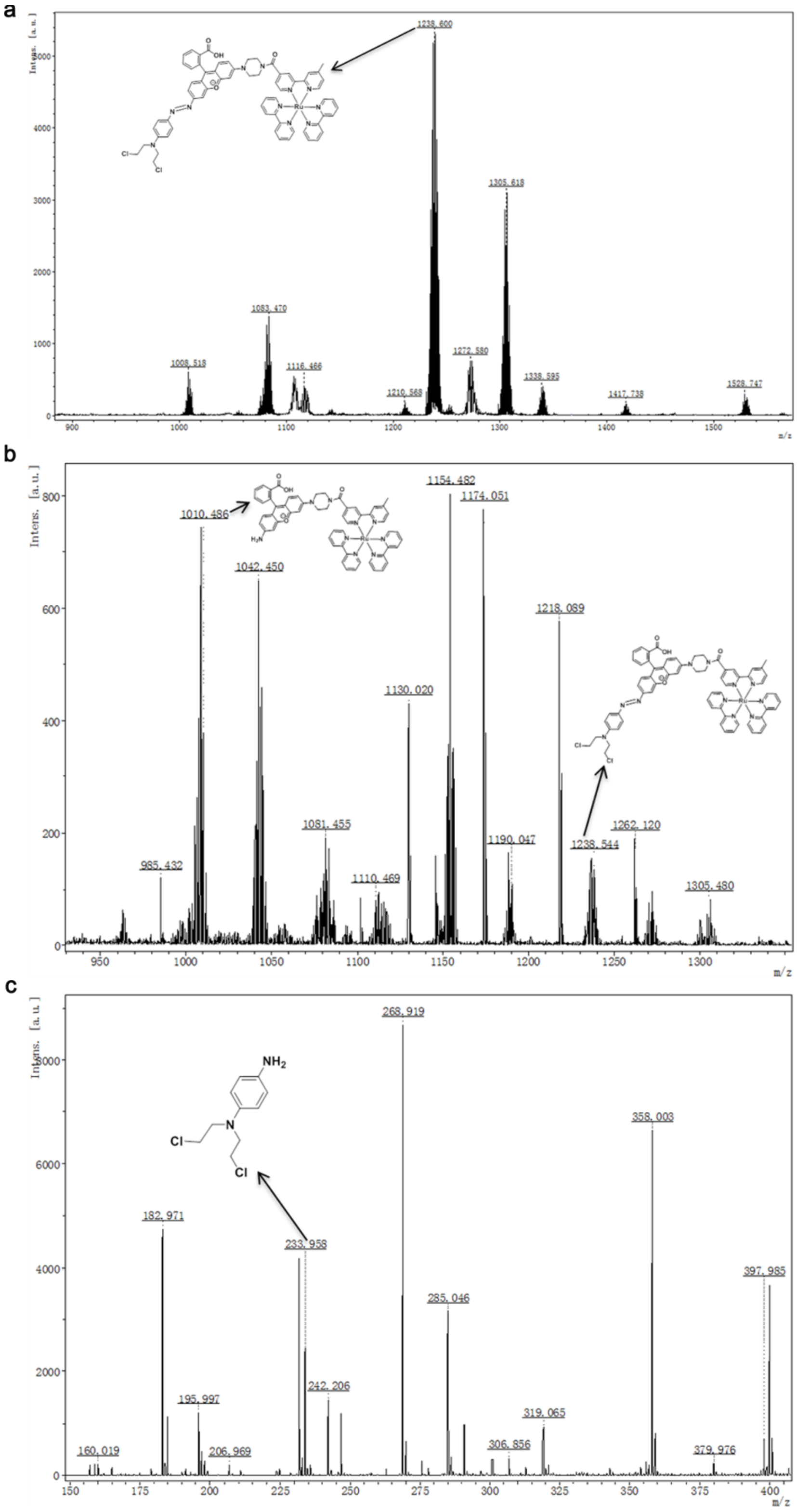

Figure S12 The MALDI-TOF/MS spectrum of **D-bpy** before (a) and after (b), (c) reduction.

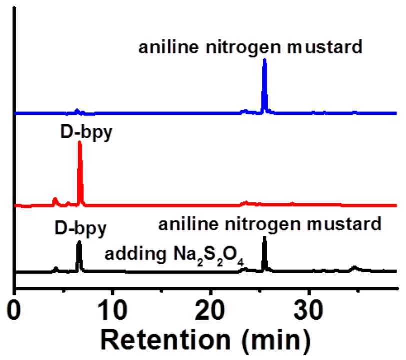

Figure S13. HPLC chromatograms in the presence of SDT ($Na_2S_2O_4$). Gradient: 5% A for 10 min, then 40% A for 20 min; A: MeCN, B: water (containing 0.2% TFA).

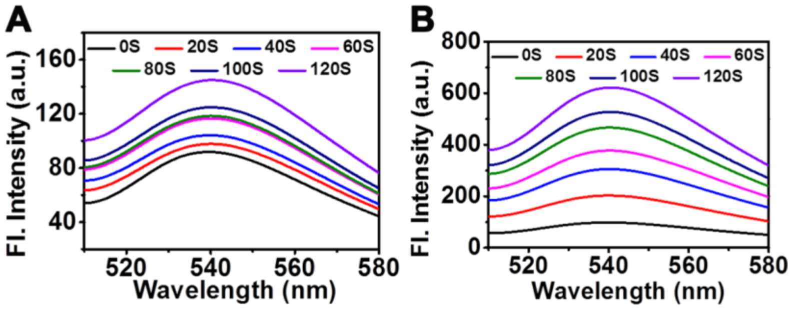

Figure S14. $^1O_2$ production of **Bpy**. Fluorescent spectra SOSG only (A) and the mixture of **Bpy** and SOSG (B) upon irradiation (irr = 450-470 nm) in MeOH.

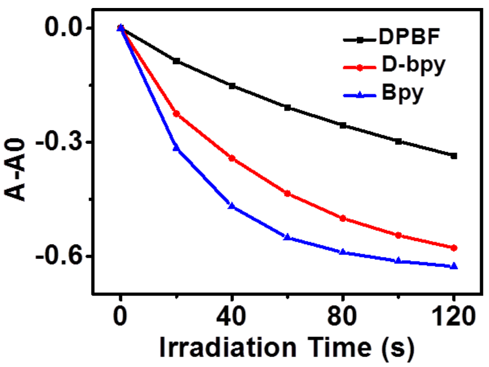

Figure S15. Measurement of $^1O_2$ production efficiency. Changes in the absorbance by DPBF at 411 nm versus irradiation time (irr = 450-470 nm) in the presence of **D-bpy** in methanol vs. $[Ru(bpy)_3]^{2+}$ as the standard.

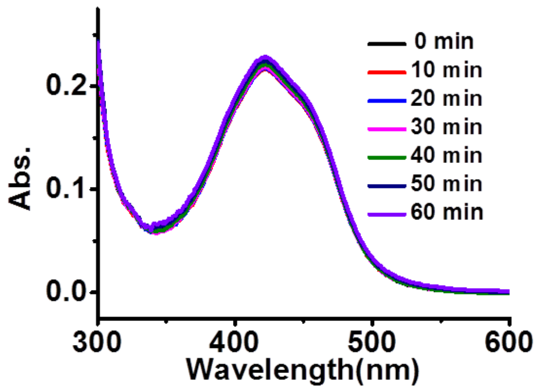

Figure S16. Light stability test of D-bpy. Absorption spectra of **D-bpy** under different light time.

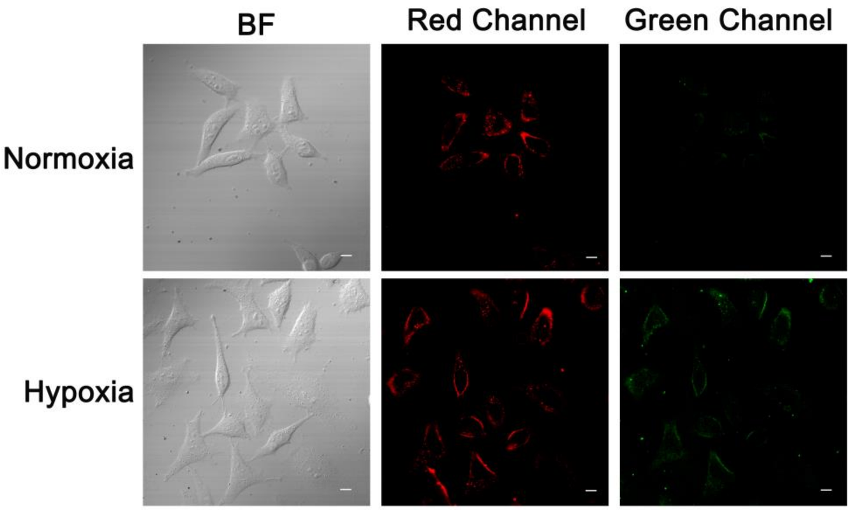

Figure S17. Confocal fluorescence microscopy at different O_2 concentrations. HeLa cells were incubated with **D-bpy** under normoxic (20% O_2) or hypoxic (1% O_2) for 5 h. Scale bars = 10 μm.

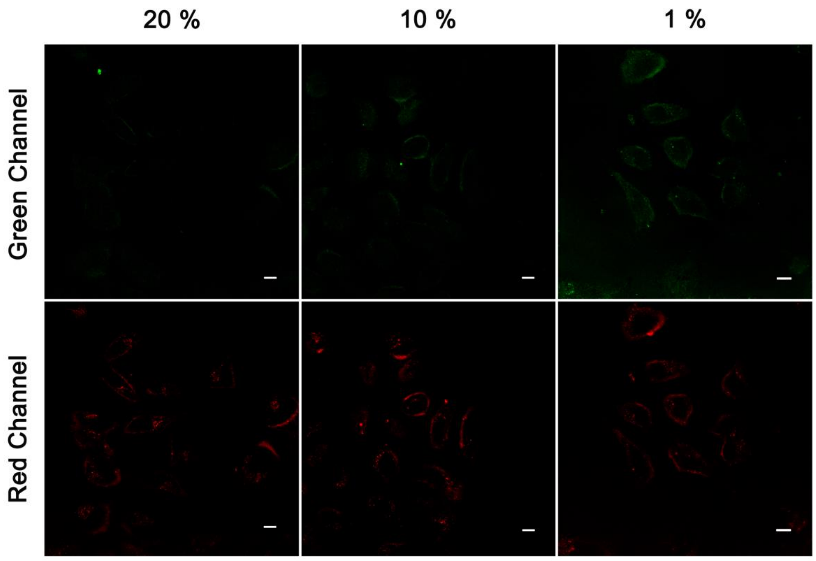

Figure S18. HeLa cells were incubated with **D-bpy** (5 μM) under different O_2 concentrations (20% O_2, 10% O_2, 1% O_2). The excitation wavelength was 488 nm, the emission wavelength was collected from 580 to 620 nm for red channel and 500 to 550 nm for green channel. Scale bars = 10 μm.

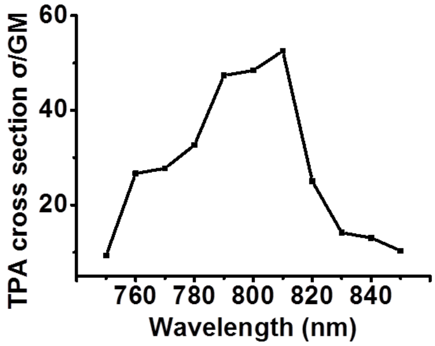

Figure S19. Two-photon properties. Two-photon absorption cross-sections of **D-bpy** (Φ = 0.01) at excitation wavelengths between 750 and 850 nm.

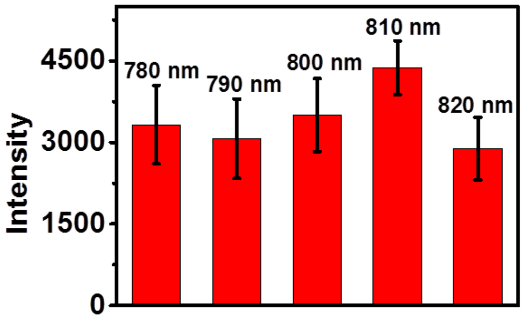

Figure S20. Two-photon properties. Mean fluorescence intensity of **D-bpy** in 4T1 cells at different TP excitation wavelengths.

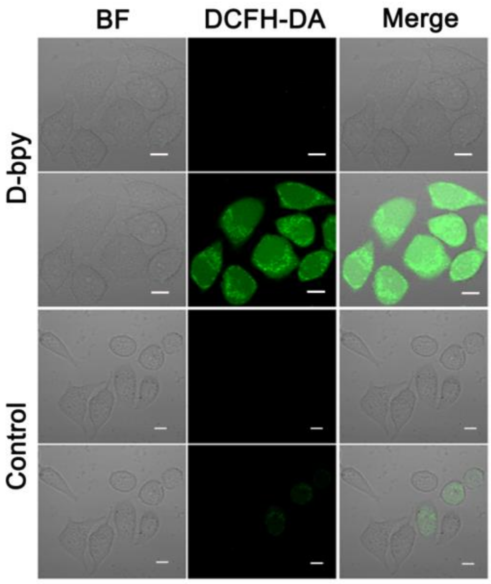

Figure S21. Intracellular $^1O_2$ production. Confocal fluorescent images of HeLa cells incubated with DCFH-DA and **D-bpy** before and after SP irradiation. Scale bars = 10 μm.

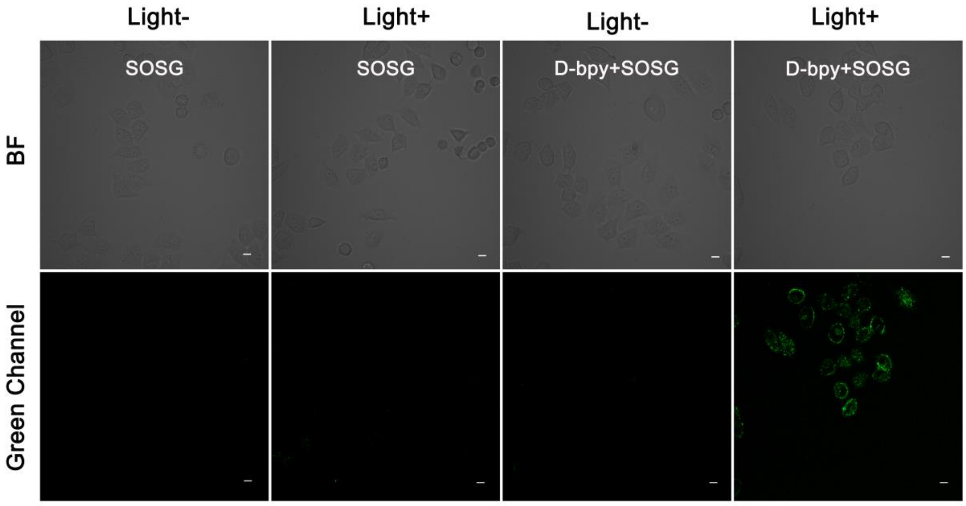

Figure S22. Confocal fluorescent images of HeLa cells incubated with SOSG and **D-bpy** before and after SP irradiation. Scale bars = 10 μm.

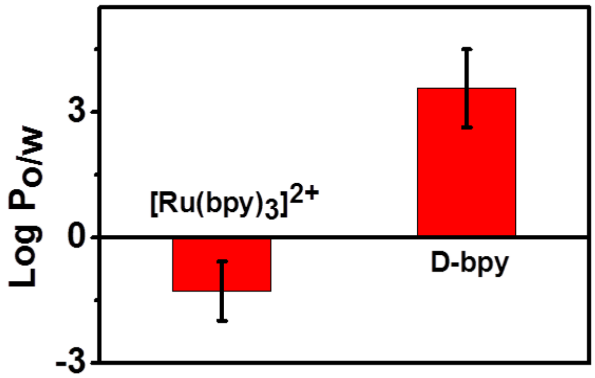

Figure S23. Octanol/water partition coefficients of $[Ru(bpy)_3]^{2+}$(**Bpy**) and **D-bpy.**

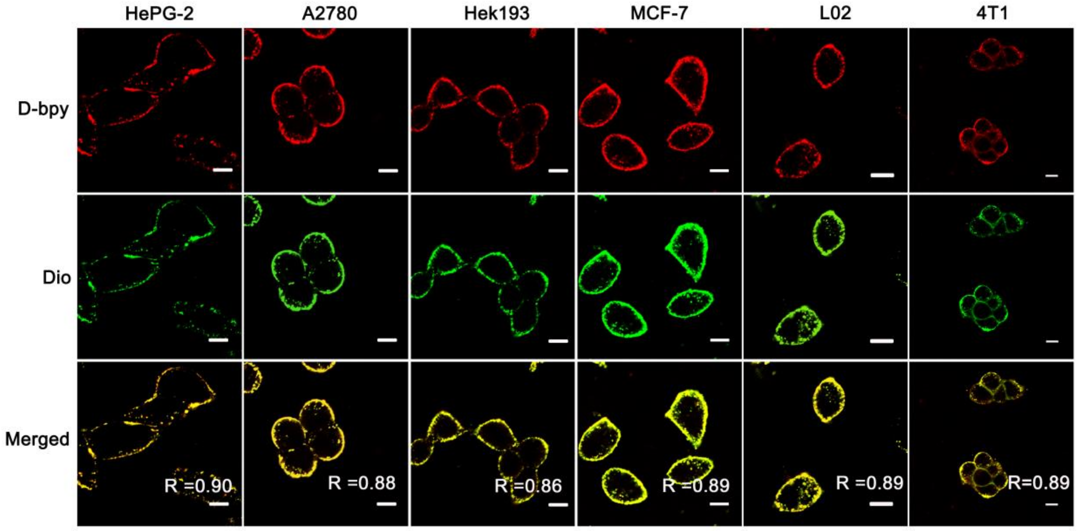

Figure S24. Co-localization images of different cells. The excitation wavelength for **D-bpy** (5 μM) and **Dio** (10 μM) were 488 nm, the emission wavelength was collected from 580 to 620 nm for **D-bpy** and 500 to 550 nm for **Dio.** Scale bars = 10 μm.

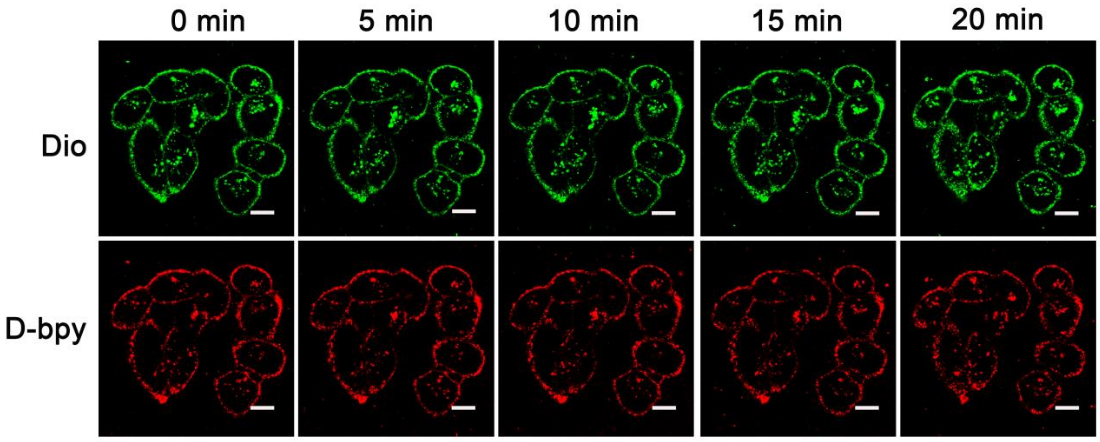

Figure S25. Real-time imaging of D-bpy and Dio in dark. Confocal images of living HeLa cells (cells were incubated with 5 μM **D-bpy** for 1 h and then incubated with 10 μM **Dio** for 40 min first) without irradiation. Scale bars = 10 μm.

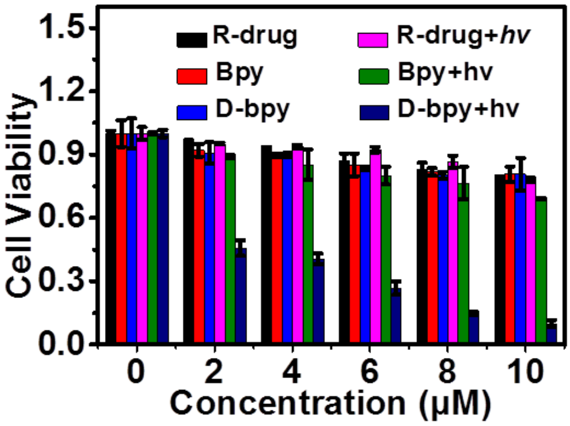

Figure S26. Cell viability of **D-bpy**, **Bpy**, **R-drug** with/without light irradiation in 4T1 cells.

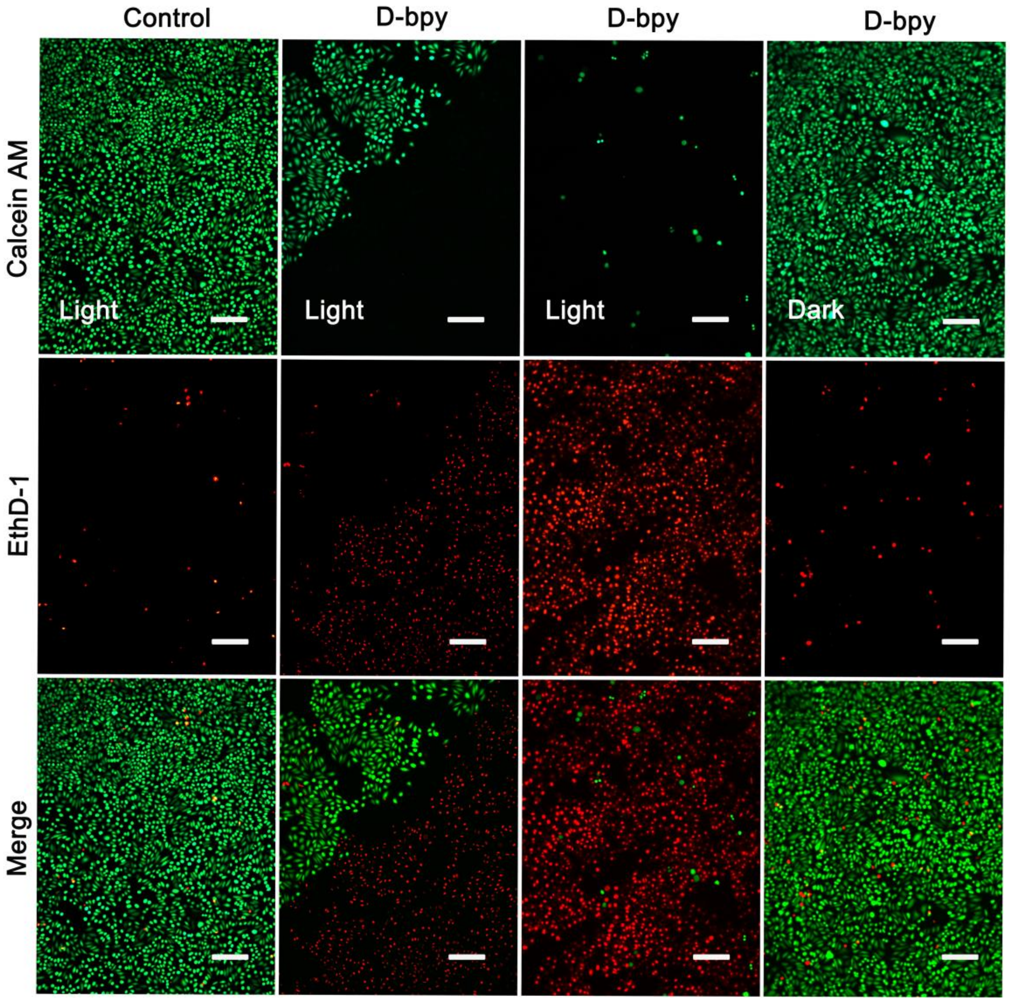

Figure S27. Live cell/dead cell staining experiments. Cell viability assay for HeLa cells incubated with **D-bpy** (5 μM) in the absence and presence of light irradiation. Scale bars = 100 μm.

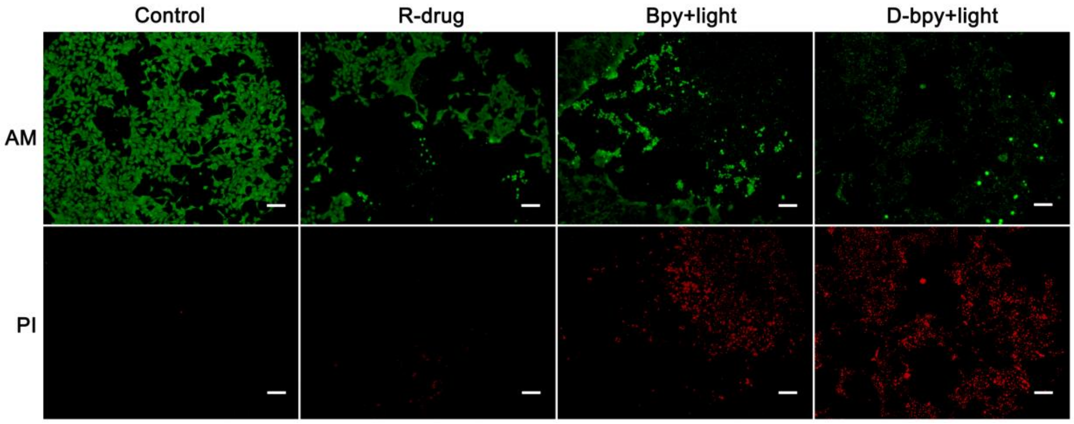

Figure S28. Cell viability assay for 4T1 cells incubated with **R-drug** (5 μM), **Bpy** (5 μM) + light and **D-bpy** (5 μM) + light under 1% O_2 conditions respectively. Scale bars = 100 μm.

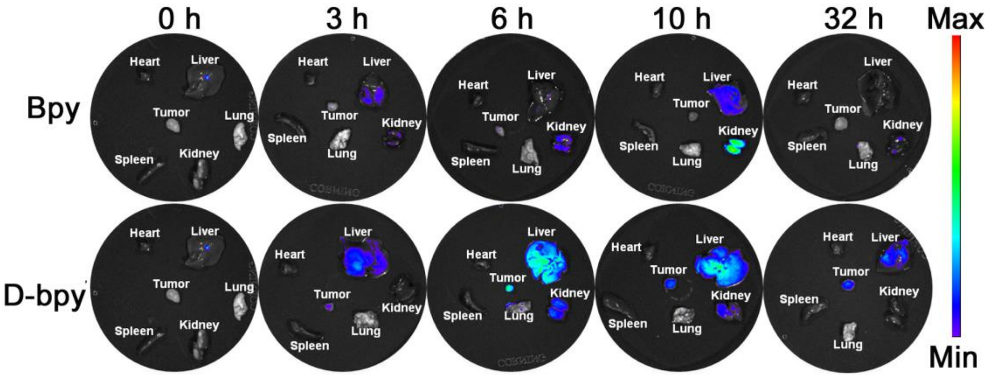

Figure S29. The main tissues and tumor tissues imaging. Fluorescence imaging of main tissues and tumor tissue from 4T1 tumor-bearing BALB/c mice after intravenous injection of **D-bpy** and **Bpy** respectively.

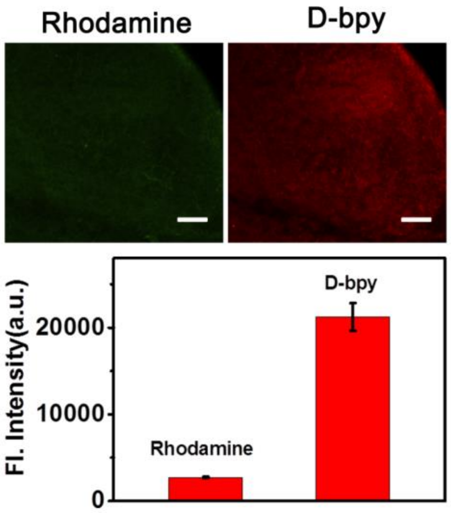

Figure S30. Tumor tissue imaging. Fluorescence images and Mean fluorescence intensity of tumor tissues from BALB/c mice with 4T1 tumor after intravenous injection of **D-bpy** for 6 h. Scale bars = 100 μm.

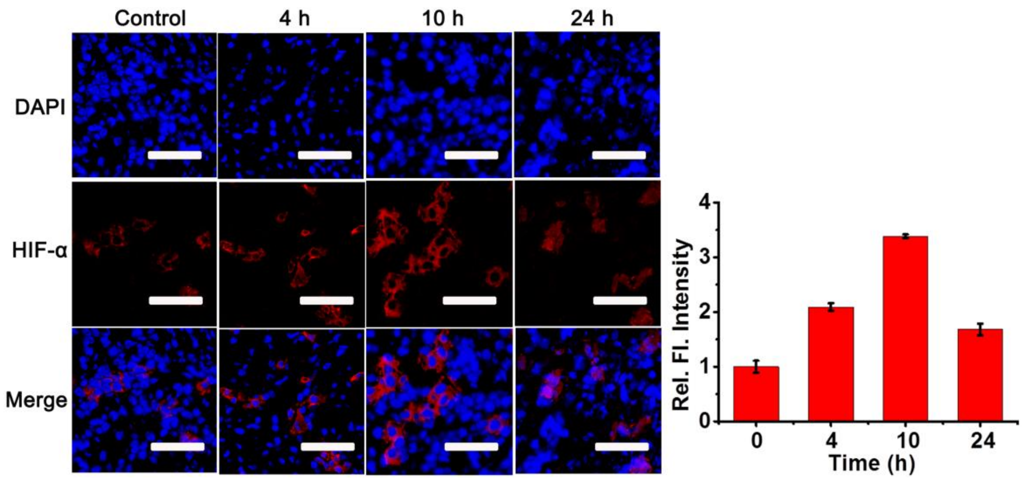

Figure S31. (A) HIF-1α staining image of 4T1 tumor tissue that was injected with **D-bpy** with light irradiation for PDT and then stopped for 4 h, 10 h, 24 h, respectively, and HIF-1α staining image of 4T1 tumor tissue that was injected with **D-bpy** without light irradiation (control). (Blue color indicated cell nucleus; red color indicated HIF-1α) (B) Relative fluorescence intensity of HIF-1α staining image from (A). Scale bars = 50 μm.

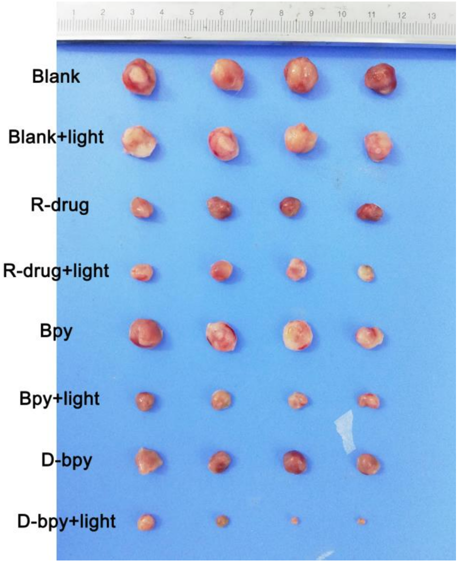

Figure S32. Tumor picture. Representative photographs from different groups (intratumoral injection).

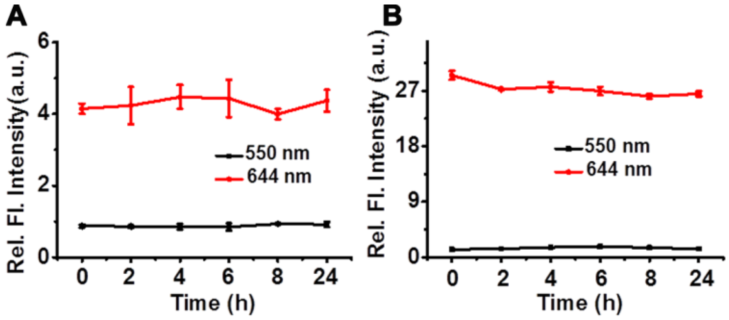

Figure S33. The stability of **D-bpy** in FBS (A) and PBS (B).

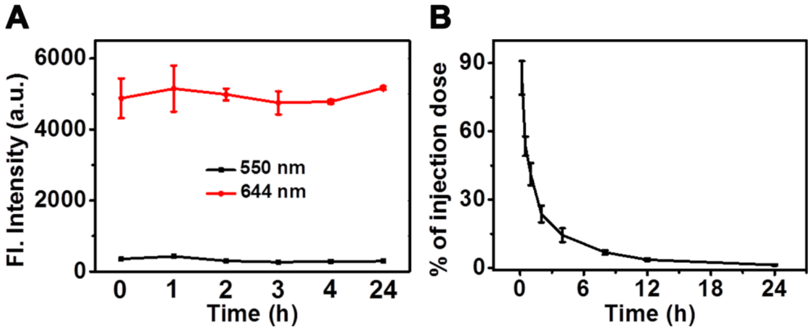

Figure S34. Stability and blood circulation test. The blood stability (A) and blood circulation (B) of **D-bpy**.

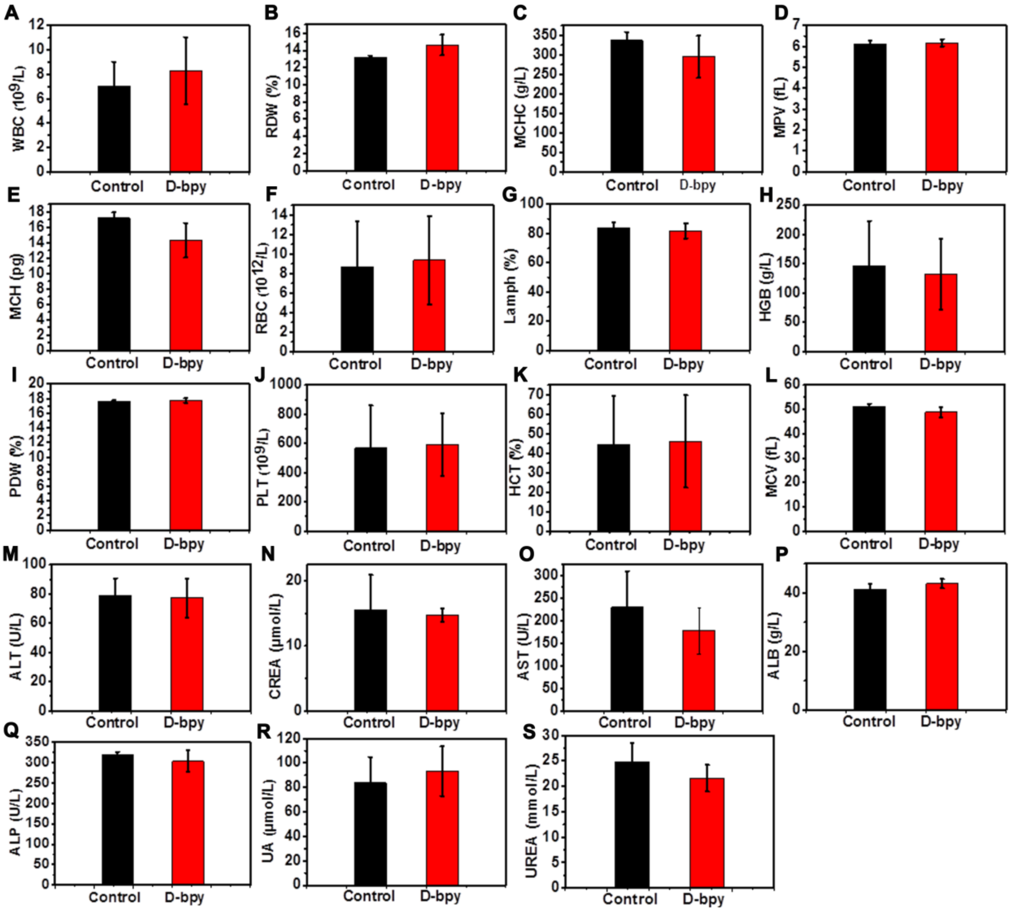

Figure S35. Hematological and biochemical analysis after i.v. injection of **D-bpy**.

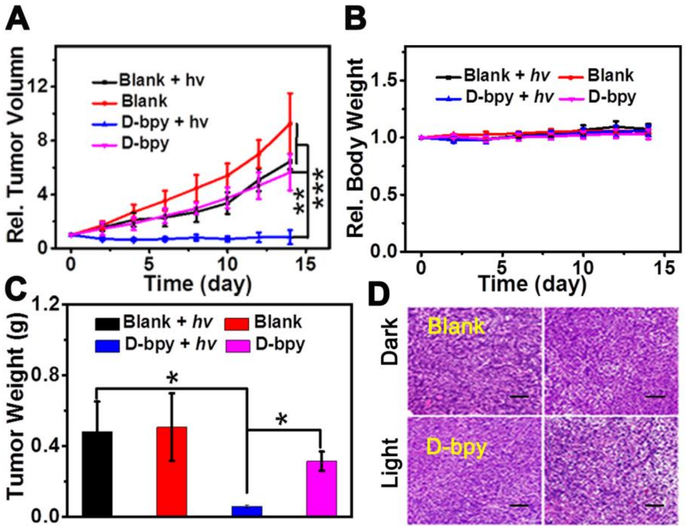

Figure S36. ***In vivo* therapeutic effect evaluation by Intravenous injection.** (A) Relative tumor volume changes of mice with different treatments (*hν* = 800 nm). (B) Tumor weight of the mice with different treatment. (C) Relative body weight of different treatments. (D) H&E staining of tumors with different treatments after PDT. Scale bars = 100 μm. *p < 0.05, **p < 0.01, and ***p < 0.001.

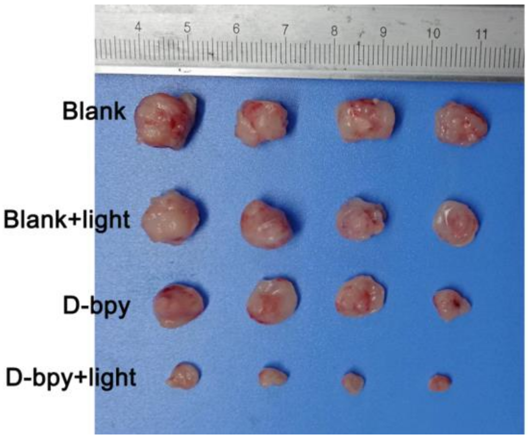

Figure S37. Tumor picture. Representative photographs from different groups (intravenous injection).

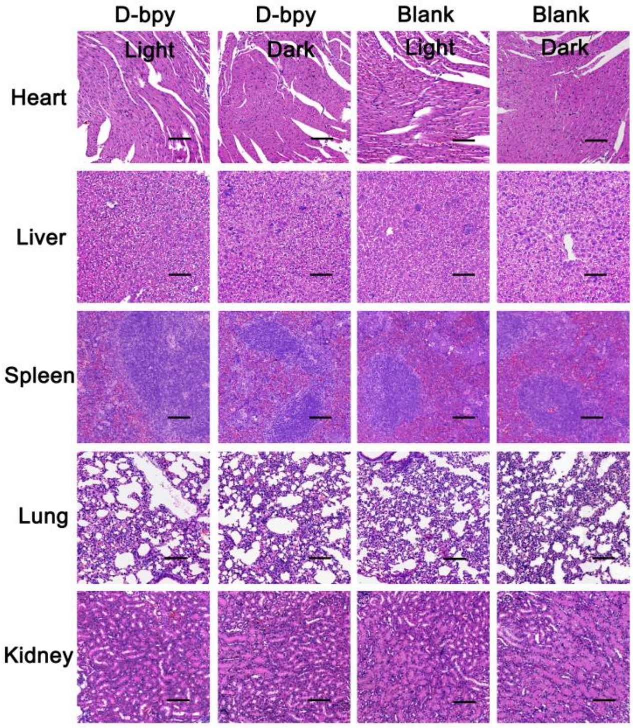

Figure S38. Histological analysis. H&E staining of the main body organs (heart, liver, spleen, lung, kidneys) with different treatments after PDT. Scale bars = 100 μm.

References

1. Cheng D, Peng J, Lv Y, Su D, Liu D, Chen M, et al. De novo design of chemical stability near-infrared molecular probes for high-fidelity hepatotoxicity evaluation in vivo. J Am Chem Soc. 2019; 141: 6352-6361.

2. Liew SS, Du S, Ge J, Pan S, Jang SY, Lee JS, et al. A chemoselective cleavable fluorescence turn-on linker for proteomic studies. Chem Commun. 2017; 53: 13332-13335.

3. Zhang W, Liu Y, Gao Q, Liu C, Song B, Zhang R, et al. A ruthenium(II) complex-cyanine energy transfer scaffold based luminescence probe for ratiometric detection and imaging of mitochondrial peroxynitrite. Chem Commun. 2018; 54: 13698-13701.

4. Peek BM, Ross GT, Edwards SW, Meyer GJ, Meyer TJ, Erickson BW. Synthesis of redox derivatives of lysine and related peptides containing phenothiazine or tris(2,2′-bipyridine)ruthenium(II). Int J Peptide Protein Res. 1991; 38: 114-123.

5. Makarov NS, Drobizhev M, Rebane A. Two-photon absorption standards in the 550-1600 nm excitation wavelength range. Opt Express. 2008; 16: 4029-4047.
